# MGS2AMR: a gene-centric mining of metagenomic sequencing data for pathogens and their antimicrobial resistance profile

**DOI:** 10.1186/s40168-023-01674-z

**Published:** 2023-10-13

**Authors:** Pieter-Jan Van Camp, V. B. Surya Prasath, David B. Haslam, Aleksey Porollo

**Affiliations:** 1 Division of Biomedical Informatics, Cincinnati Children’s Hospital Medical Center, Cincinnati, OH 45229 USA; 2 Department of Pediatrics, University of Cincinnati, Cincinnati, OH 45267 USA; 3 Division of Infectious Diseases, Cincinnati Children’s Hospital Medical Center, Cincinnati, OH 45229 USA; 4 Center for Autoimmune Genomics and Etiology, Cincinnati Children’s Hospital Medical Center, Cincinnati, OH 45229 USA

**Keywords:** Antimicrobial resistance, Graphical fragment assembly, Metagenomics, Microbiome

## Abstract

**Background:**

Identification of pathogenic bacteria from clinical specimens and evaluating their antimicrobial resistance (AMR) are laborious tasks that involve in vitro cultivation, isolation, and susceptibility testing. Recently, a number of methods have been developed that use machine learning algorithms applied to the whole-genome sequencing data of isolates to approach this problem. However, making AMR assessments from more easily available metagenomic sequencing data remains a big challenge.

**Results:**

We present the Metagenomic Sequencing to Antimicrobial Resistance (MGS2AMR) pipeline, which detects antibiotic resistance genes (ARG) and their possible organism of origin within a sequenced metagenomics sample. This in silico method allows for the evaluation of bacterial AMR directly from clinical specimens, such as stool samples. We have developed two new algorithms to optimize and annotate the genomic assembly paths within the raw Graphical Fragment Assembly (GFA): the GFA Linear Optimal Path through seed segments (GLOPS) algorithm and the Adapted Dijkstra Algorithm for GFA (ADAG). These novel algorithms improve the sensitivity of ARG detection and aid in species annotation. Tests based on 1200 microbiome samples show a high ARG recall rate and correct assignment of the ARG origin. The MGS2AMR output can further be used in many downstream applications, such as evaluating AMR to specific antibiotics in samples from emerging intestinal infections. We demonstrate that the MGS2AMR-derived data is as informative for the entailing prediction models as the whole-genome sequencing (WGS) data. The performance of these models is on par with our previously published method (WGS2AMR), which is based on the sequencing data of bacterial isolates.

**Conclusions:**

MGS2AMR can provide researchers with valuable insights into the AMR content of microbiome environments and may potentially improve patient care by providing faster quantification of resistance against specific antibiotics, thereby reducing the use of broad-spectrum antibiotics. The presented pipeline also has potential applications in other metagenome analyses focused on the defined sets of genes.

Video Abstract

**Supplementary Information:**

The online version contains supplementary material available at 10.1186/s40168-023-01674-z.

## Background

Antibiotic-resistant bacteria are a global problem causing millions of deaths worldwide [1, 2]. Early detection of emerging human pathogens and rapid evaluation of their antimicrobial resistance (AMR) profile are of vital importance. Such detection and evaluation can guide the choice of an effective antibiotic (AB) treatment regimen and can prevent the development of novel AMR [3]. A common way for bacteria to develop AMR is by acquiring antibiotic resistance genes (ARG) [4]. One of the most comprehensive, curated lists of ARG is maintained by the NCBI Bacterial Antimicrobial Resistance Reference Gene Database [5]. Bioinformatics tools leveraging the high-throughput sequencing (HTS) data from bacterial isolates are already able to detect ARG presence and subsequently predict their AMR [6]. Deployment of such tools in clinical settings can potentially shorten the time needed to obtain antibiograms compared to the current practice of laboratory culturing and AB testing. Faster antibiograms will make therapy more effective and improve patient outcomes [7, 8].

Clinical samples containing just a single bacterium are mostly limited to infections of otherwise sterile compartments of the body, such as blood or cerebral spinal fluid. The other body sites, however, such as the intestines, lungs, or skin, are populated with a mix of various microbes (collectively called a *microbiome*) of which the bacterium causing an infection is only one [9]. Evaluation of these samples requires the clinical laboratory to first isolate a suspected pathogen from a mixed population and then perform antibiotic susceptibility testing; a process that may require several days. Hence, broad-spectrum antibiotics are often used to treat these infections while awaiting identification and characterization of the infecting organism, though sometimes a therapy can be tailored when PCR-based detection of pathogens or some specific ARG is available [10–12]. Furthermore, culture-based methods are sometimes incapable of separating pathogens from other non-pathogenic organisms, in which case antibiotic susceptibility of the infecting organism cannot be determined using traditional methods.

Identifying a pathogen amongst the commensal microbes in the background and determining its specific AMR profile are the next logical steps, but it comes with a set of challenges. High-throughput metagenomics sequencing (MGS) has to provide sufficient coverage and depth to detect ARG and its origin. Furthermore, the metagenomic sequencing reads are generally short pieces of DNA—dozens to several hundred base pairs (bp) long—that originate from any genomic regions, including those highly conserved across different strains (pathogenic and commensal). Another challenge of the MGS analysis, more specific to ARG detection, is that many ARG are located on plasmids, i.e., mobile genomic elements that exist outside of a bacterial genome and can be highly similar between species or shared across species (e.g., through conjugation) [13]. Furthermore, a single bacterium can have multiple copies of a plasmid or even contain different plasmids [14].

In this work, we present MGS2AMR—a computational pipeline for the AMR evaluation of a microbiome sample based on high-throughput sequencing data. MGS2AMR detects ARG and suggests their origin. The results of the pipeline can be visualized and analyzed with an associated tool called MGS2AMR Explorer. We further demonstrate a potential clinical application of the pipeline by using machine learning to detect the presence of potential pathogens within the intestinal microbiome and evaluating their geno- and phenotypical AMR, which may provide the clinician with advance notice of the presence and antibiotic resistance profile of an organism causing impending invasive infection. Such a workflow may improve clinical practice by providing the AMR information early in the infection phase before complications, such as sepsis, arise.

The source code and latest release are available at https://github.com/pieterjanvc/mgs2amr.

## Methods

**Fig. 1:**
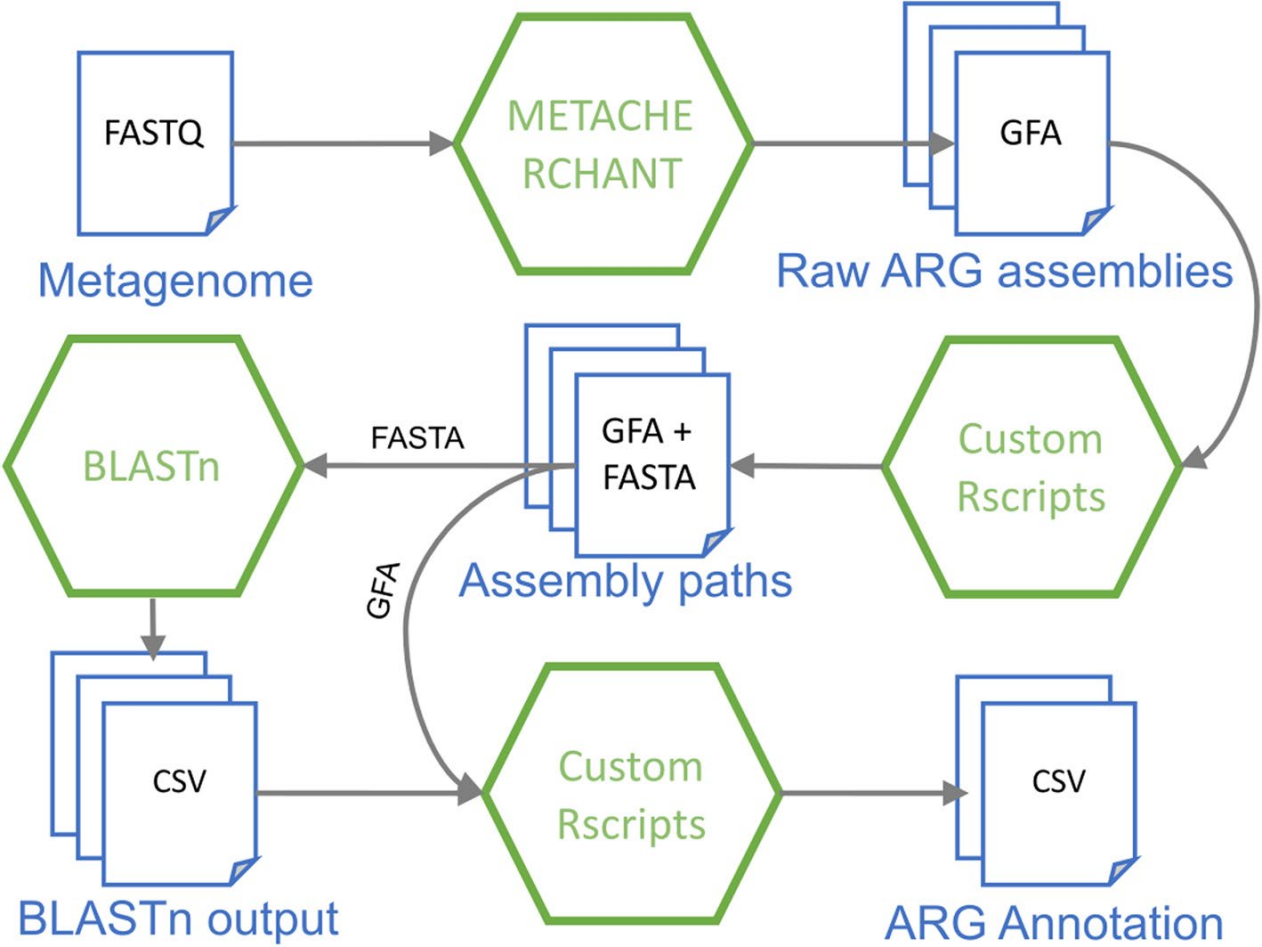
A flowchart of the MGS2AMR pipeline. The MGS2AMR pipeline accepts metagenomic sequencing (MGS) data as short-read sequencing files in the FASTQ format. The MGS data is fed to MetaCherchant that makes genomic assembly using ARG sequences as seeds. The resulting raw assembly (GFA) files are subsequently processed to reconstruct the ARG and to score the assembly paths. These scores, in conjunction with the BLASTn homology search, are used to annotate the identified ARG with respect to the origin (bacterial species or plasmid). Rectangles represent data files, hexagons—software employed. File formats: FASTA, FASTQ, GFA Graphical Fragment Assembly, CSV comma-separated values

Figure 1 provides a high-level overview of the proposed pipeline. MGS2AMR starts the search for known ARG within the MGS data using MetaCherchant [15] that makes alignment-free assemblies seeded by a set of reference genes. The identified ARGs are filtered by the quality of the assembly. For every retained ARG, the assembled surrounding genomic regions are evaluated to identify alternative paths over the sequence segments. These segments are then annotated and scored through the homology search against a bacterial nucleotide database, e.g., retrieved from NCBI, using BLASTn [16]. Finally, the assembly graphs and BLASTn results are combined to score the recovered ARG and link them with specific bacteria or plasmids of origin. The raw output from the MGS2AMR pipeline can be further processed depending on the application. Here, we demonstrate a specific application of MGS2AMR by using its output to predict AMR to a set of commonly used AB within clinically important bacteria. The following sections will detail the tools and approaches used at every step.

### Metagenomic sequencing analysis

#### ARG detection

The MGS2AMR pipeline starts with detecting known ARG in the MGS data using MetaCherchant [15]. This alignment-free metagenomics assembler makes assembly from a set of user-specified DNA seed sequences. The ARG sequences used as seeds were retrieved from the curated NCBI Bacterial Antimicrobial Resistance Reference Gene Database (NCBI Accession ID: PRJNA313047). As of June 2021, this database contained information on 5804 ARG across all classes of AMR and bacterial species. If a seed is at least partially detected in the MGS, MetaCherchant initiates the assembly of the genomic region surrounding the seed. MetaCherchant is used with default settings, except for an imposed extension limit of 2000 nucleotides (nt) and chunk length of 250 nt to accelerate the assembly and limit the size of the output files.

**Fig. 2:**
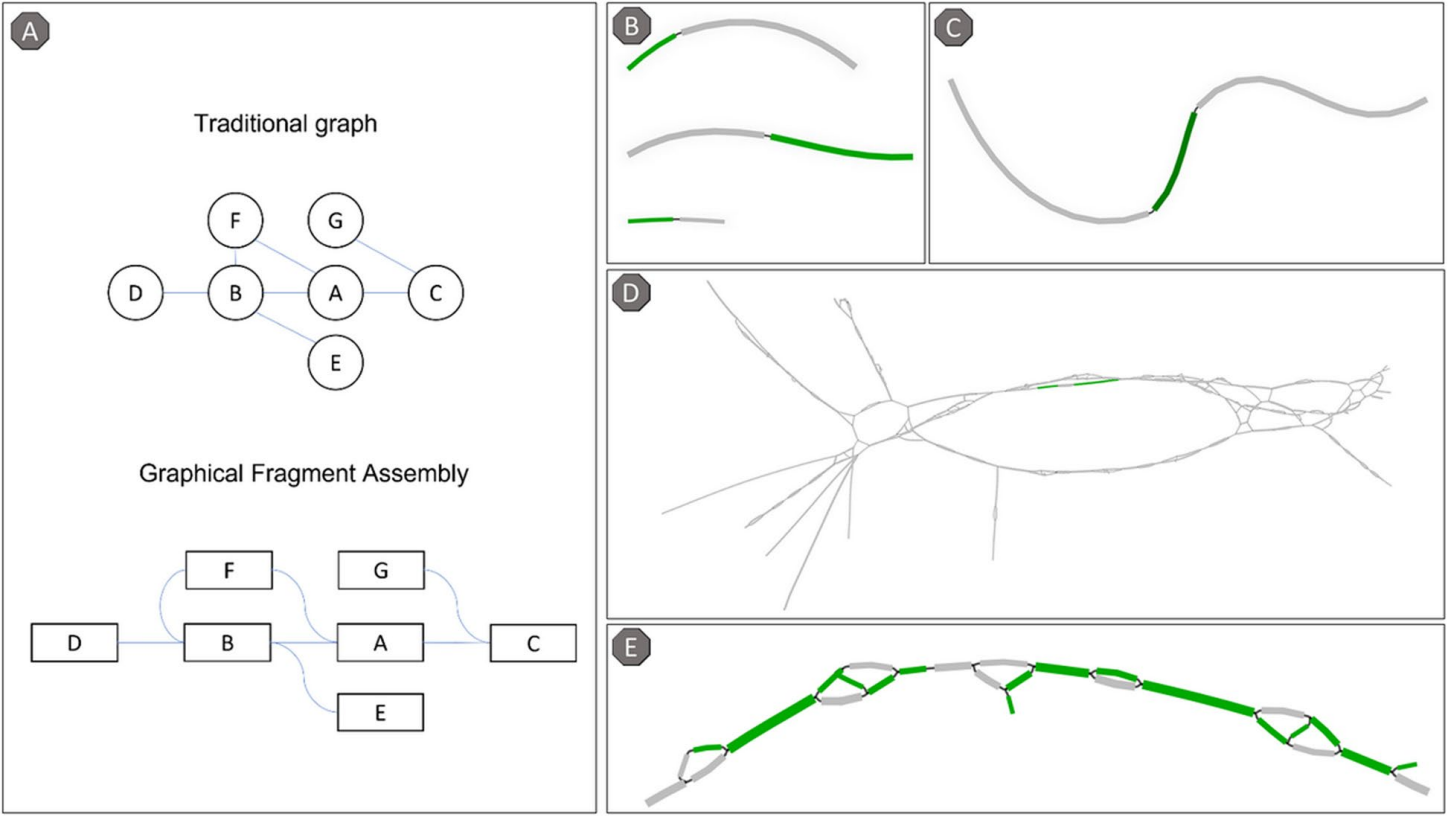
Graphical Fragment Assembly. **A** Comparison between a traditional graph and a GFA. Circles represent nodes, rectangles**—**segments, lines are connections. **B** Incomplete assembly of ARG (not all fragments are linked). **C** Simple linear assembly from an ARG seed. **D** Complex branched assembly around ARG. E Zoom-in on the fragmented seed ARG assembly from panel **D**. Segments originated from an ARG seed are highlighted green. Gray segments represent anextension of the assembly beyond the seed sequence. Here and in the following figures, GFA are visualized using the Bandage tool [18]

MetaCherchant generates graphical fragment assembly (GFA) files that list unitigs, linear sequence subgraphs of the overall graph. GFA is a type of graph that considers segments instead of nodes (Fig. 2A). Unlike nodes, a segment is a piece of DNA with 3′ and 5′ ends and therefore has direction. The GFA file specifies which ends of neighboring segments are connected and how large the overlap is [17].

MetaCherchant generates a separate GFA for each ARG detected in the MGS data. The partially present seeds yield either multiple disconnected segments (Fig. 2B) or the seed segments bridged by non-seed segments (Fig. 2D–E). Ideally, GFA should represent a simple linear extension of ARG into its surrounding genomic regions (Fig. 2C). Practically, however, GFA is a complex network of diverging paths consisting of many smaller segments (Fig. 2D) due to the following possible reasons. (1) The ARG is (partially) present in multiple species within the metagenome, which results in the different surrounding genomic regions. (2) Other unrelated genomic regions in the metagenome share similarities with the ARG or immediate surroundings. (3) Sequencing errors or other technical imperfections result in lower quality reads preventing correct assembly. (4) The bacterium is in low abundance (i.e., insufficient sequencing coverage) that makes assembly incomplete.

#### ARG assembly refinement

**Fig. 3:**
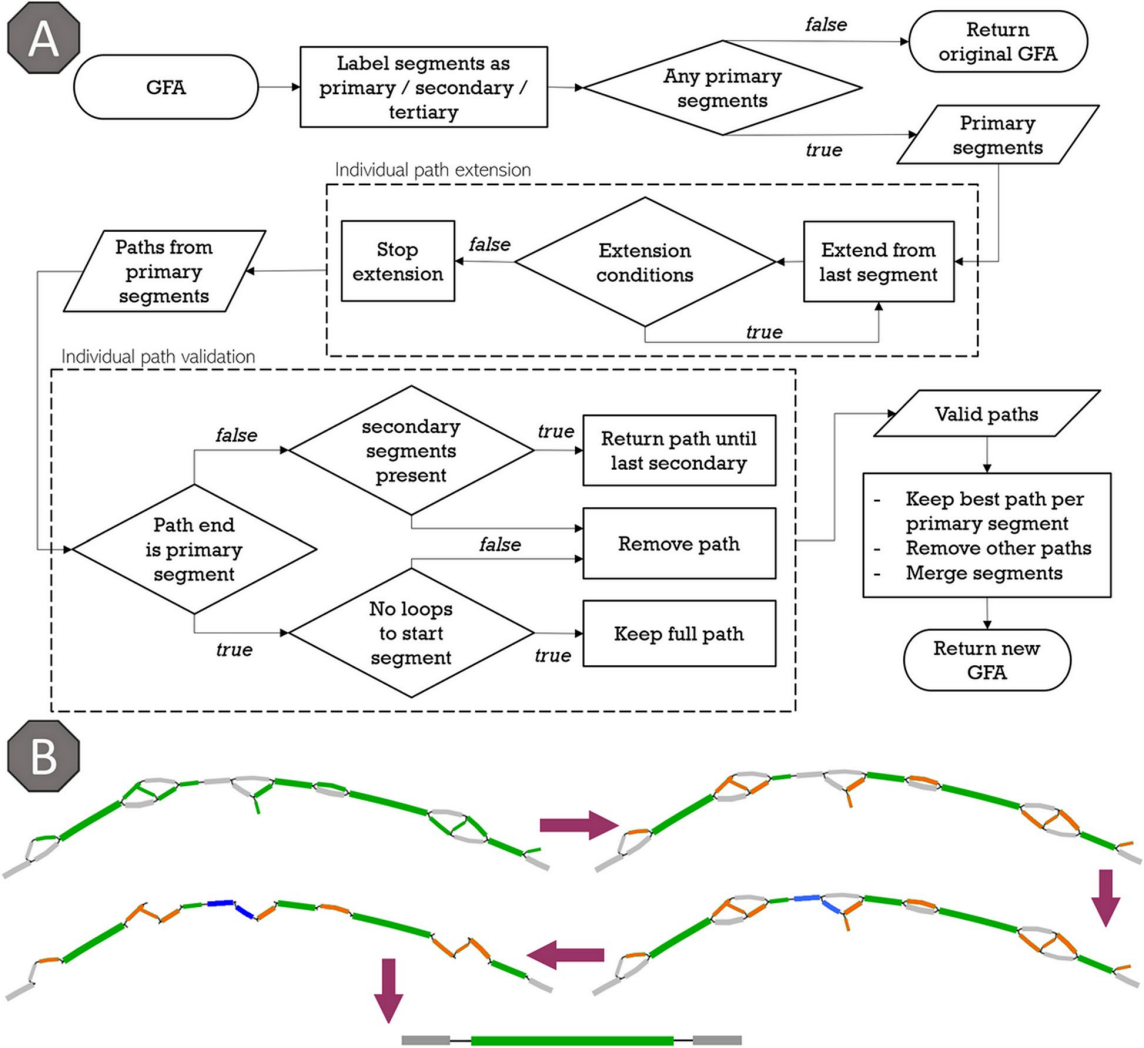
GLOPS algorithm. **A** Flowchart of the algorithm. **B** Illustration of the work of GLOPS. Green segments are primary seed segments (non-bypassable), orange—secondary seed segments (bypassable), and gray—tertiary segments (non-seed). Blue segments are tertiary segments that are part of the final path

We developed the GFA Linear Optimal Path through Seed segments algorithm (GLOPS, Fig. 3A) to combine discontinuous seed segments (e.g., Fig. 2E) into one contig to reconstruct the full ARG sequence. First, the non-bypass and bypass seed segments are assigned as *primary* (*S*^*p*^, marked green in Fig. 3B) and *secondary* (*S*^*s*^, orange), respectively. All non-seed segments are *tertiary* (*S*^*t*^, gray). Next, paths (*P*) over adjacent segments are extended from all primary segments in both directions until one of the following four criteria is met (Eq. 1): (1) Another primary segment is encountered. (2) The end of the graph is reached. (3) The path over secondary and tertiary segments exceeds the maximum length (*L*_*max*_, set to 800 bp in this work). (4) The number of alternative paths exceeds the limit (set to 5000 per primary segment in this work). The latter is critical to prevent computer memory overload while storing a high number of branching paths in case of poor-quality assemblies. The path search stops when all primary and secondary segments are found and linked.

1 $$\begin{array}{c}P=\left({S}_{k}^{p}\vee \varnothing ,\left\{\left\{{S}_{U}^{s},{S}_{V}^{t}\right\}, {S}_{i}^{p},\left\{{S}_{X}^{s},{S}_{Y}^{t}\right\}\right\},{S}_{l}^{p}\vee \varnothing \right)\\ \sum \left|\left\{{S}_{U}^{s},{S}_{V}^{t}\right\}\right|\le {L}_{max} \wedge \sum \left|\left\{{S}_{X}^{s},{S}_{Y}^{t}\right\}\right|\le {L}_{max}\\ U\cap X=\varnothing \wedge V\cap Y=\varnothing ,\end{array}$$

where *P* is a path encompassing the *i*th primary segment *S*^*p*^_*I*_ surrounded by the *U*, *V*, *X*, and *Y* sets of adjacent secondary and tertiary segments *S*^*s*^ and *S*^*t*^, respectively, and terminating at *k*th and *l*th primary segments (*S*^*p*^_*k*_ and *S*^*p*^_*l*_) or at the end(s) of the graph. Path weight is defined as the total number of k-mers, mapped to the given path, normalized to the total length of the path. All lengths hereinafter refer to the number of nucleotides in the sequence.

Then, the most optimal paths between primary segments are chosen based on the following criteria. (1) If there are paths from one primary segment to multiple other primary segments, the shortest path is chosen, provided the connecting primary segment is not an end segment. If primary segments are end segments, the longest path is chosen. (2) If there are multiple paths between two primary segments, the one with the longest secondary segment length is retained, keeping the path with the highest weight in case of ties.

In special cases, such as loops, the connection between primary segments with the longest distance is dropped to ensure a linear path. Loops can occur at low-quality assemblies. Also, if a path does not end at a primary segment (e.g., reached the end of the graph), the last encountered secondary segment is considered as the end of the path.

Lastly, all segments (and their extensions) between the start and end of the final path, but not part of it, are pruned resulting in a linear path that represents the reconstructed seed ARG. Isolated seed fragments may still be present in the graph if not all primary segments are connected (e.g., the gap is too large, or no path is found). Each reconstructed seed sequence is then evaluated on coverage and sequence identity to the original ARG to be reported in the final output. In some cases, the full assembly cannot be completed, either due to a lack of coverage of the seed or because of a false positive match. This results in multiple small fragmented assemblies (Fig. 2B). An assembly is labeled as fragmented when the longest primary segment either has no extension or extends on one side only. Of note, tertiary segments below 100 bp are ignored while assessing assemblies. When fragmented, the coverage and sequence identity of the ARG are estimated simply by summing all seed segment lengths.

At the end, GLOPS generates a table of all detected ARG with corresponding coverage, sequence identity to the seed, weight, and type (fragmented or not).

#### Filtering false positive and duplicate ARG

Any ARG with coverage below 25% is removed as a false positive. On the other hand, the ARG reference database contains many genes that have high sequence similarity or represent subtypes (e.g., alleles) of the same ARG, such as the family of OXA β-lactamases [19]. Similar genes will generate nearly identical assemblies. Therefore, it is necessary to remove duplicates and choose representatives for downstream analysis.

Assuming a particular ARG is present in a given metagenome, its close orthologs from the ARG reference database will also yield assemblies resulting in similar GFA. Isomorphism detection in graph theory is a problem that has no solution in polynomial time [20]. However, since GFA is a special type of graph (Fig. 2A), a fast approximation is achievable. It is very unlikely that two genomic regions of different origins within the metagenome would assemble in the exact same way, i.e., generate an identical list of segments. If two GFA share the exact same list of non-seed segments, one can assume these GFA are identical without the need to compare the layout of links between them. This simple list comparison identifies all duplicates in linear time.

This approach does not work when the GFA are not identical, i.e., there are minor differences in segments. In this case, the identity of segments between GFA is assessed using the *calc_distmx* function from the Usearch package [21]. This comparison is more computationally intensive and is therefore limited to the seed sequence and the immediately adjacent segments. If all segments in one GFA share sequence similarity over 90% to the corresponding segments in the other GFA, both assemblies are considered identical for MGS2AMR. For all GFA found identical, the seeds with the highest coverage, sequence identity, and depth are kept for further analysis and the rest are removed as duplicates.

### Evaluating the ARG genomic context

#### Pathfinding in genomic extensions of seed genes

After reconstructing and culling the ARG seeds in the GFA, MGS2AMR evaluates the assembly of the surrounding non-seed genomic regions. The goal is to identify paths in the graph that represent bacterial genomes from which the ARG originated. For simple GFA (e.g., Fig. 2C), this is straightforward as there is only one path in either direction extending from the seed. However, in complex graphs (e.g., Fig. 2D), the enumeration and evaluation of all possible paths is a hard combinatorial problem. To tackle this, several steps are taken to retain only the most probable paths.

**Fig. 4:**
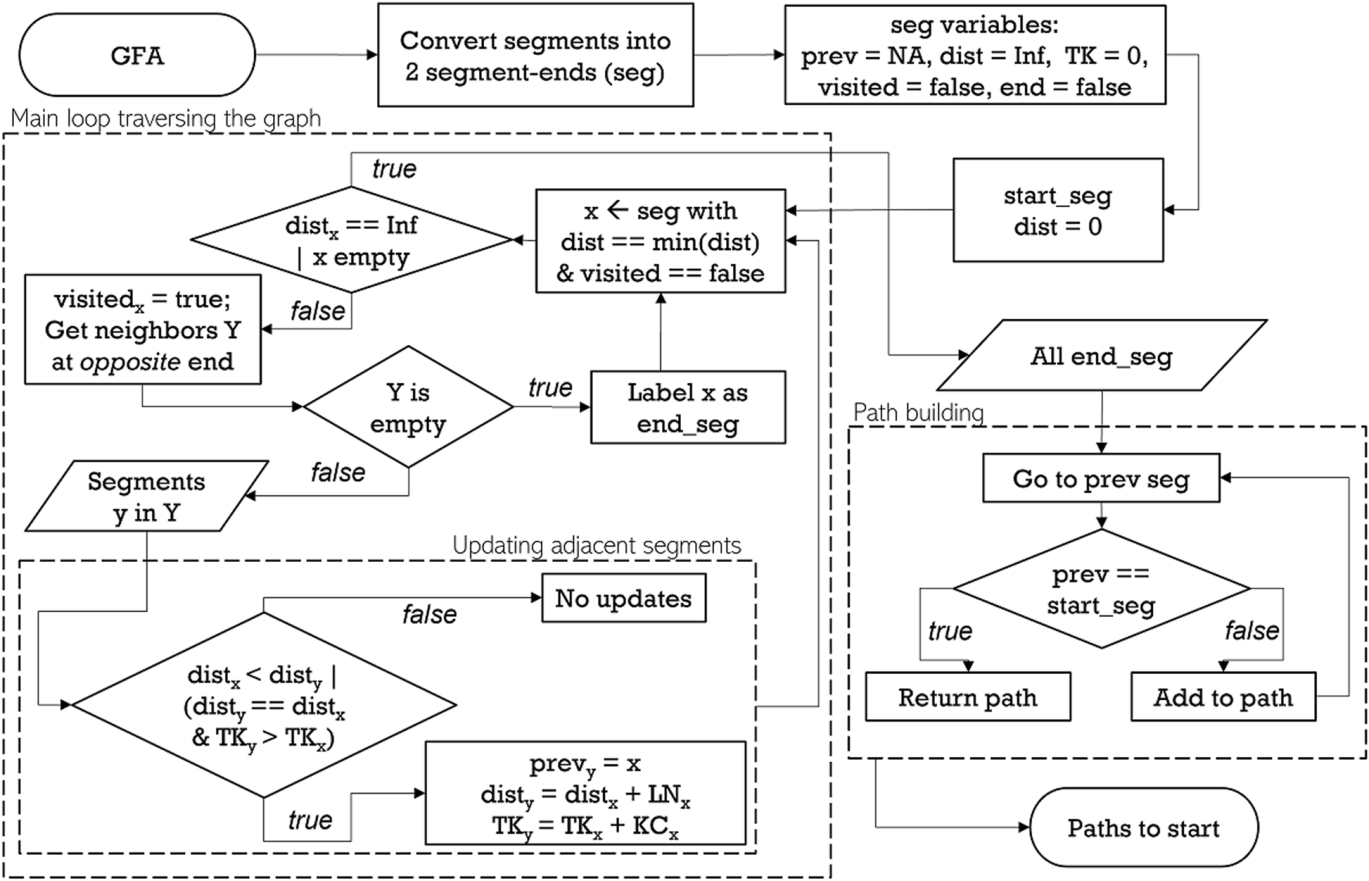
Adapted Dijkstra Algorithm for GFA (ADAG). All paths emanating from a given segment (i.e., the reconstructed seed segment) are enumerated, whilst keeping track of the distance traveled (in bp) and the total k-mer count. Since segments have two directions, the path search is performed from both the 3′ and 5′ ends, also keeping track of the orientation in which segments are traversed. The search continues until all segments that can be reached have been visited or when a specified limit (distance from the start segment or number of iterations) is exceeded. Once finished, the shortest paths from the end segments are found by backtracking through the graph to the starting segment. Variables: seg = the 3′ and 5′ segments ends (i.e., each segment has 2 orientations); prev = previous segment in shortest path; dist = distance from current segment to start; TK = total k-mer count from current segment to seed; visited = segment has been visited by algorithm; end = end-segment (true/false); LN = segment length; KC = segment k-mer count; NA = Not Available; Inf = Infinity

First, the search is limited to all shortest paths from end segments to the seed segment. An end segment has an adjacent segment on one side only (e.g., GFA segments C, D, E, and G in Fig. 2A). To identify all shortest paths from these end segments, we developed a new Adapted Dijkstra Algorithm for GFA (ADAG, Fig. 4). ADAG is based on the classical Dijkstra’s algorithm [22] for finding the shortest paths and has been adapted to accommodate the differences in graph traversal in GFA, where the direction of segments (DNA sequences) is important. For example, let us consider the path from E to A in Fig. 2A. In the traditional graph, the shortest path is (E,B,A), while it is (E,B,F,A) in the GFA. It is not possible to walk from B to A, as E and A are connected to the same side of B. Similarly, there is no path from G to A in the GFA, whereas (G,C,A) is traversable in the traditional undirected graph. Some paths like (D,B,A) are valid in both graphs.

Once ADAG is complete, all paths in the GFA are evaluated for loops, which can form because segments have two orientations. A loop is defined when a segment is used twice in the same path (in sense and antisense direction). These loops are artifacts of imperfect DNA assembly (e.g., sequencing errors, repeats, overlapping regions inter- or intra-genomic, etc.) and need to be resolved. Additional file 1: Figure S1 shows the different types of loops and how paths are corrected when loops occur.

**Fig. 5:**
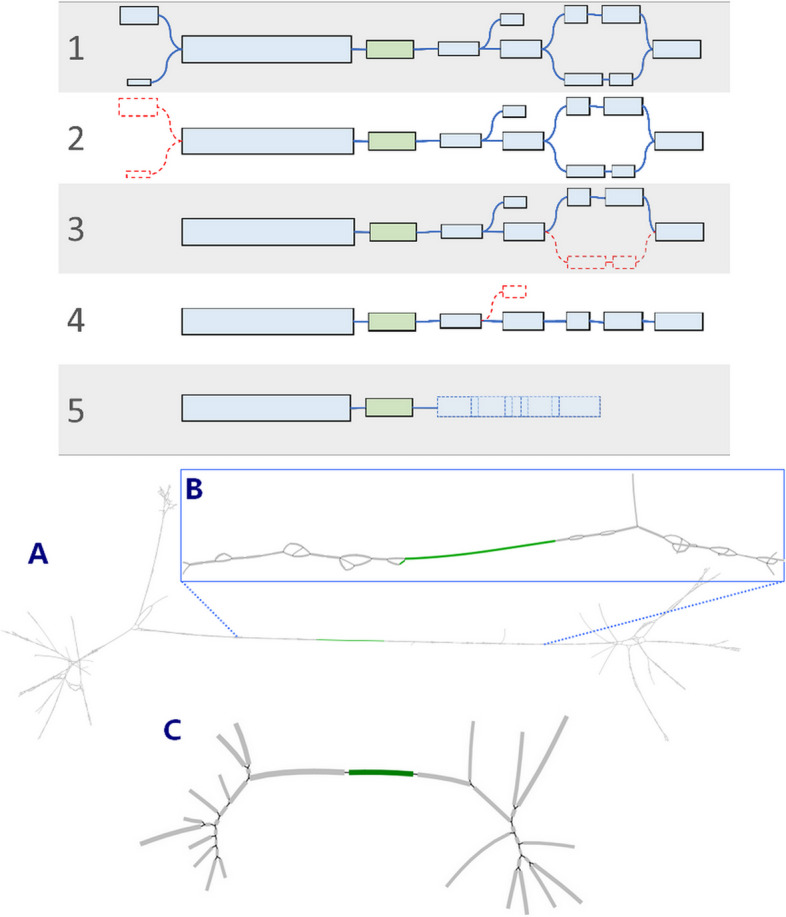
Illustration of ADAG operation. Top panel. Schematic representation of best path retention in GFA. The green segment is the seed, and blue/gray are the sequence assembly extensions. 1. Example of a GFA before path detection. 2. Removal of segments beyond the maximum distance from the seed. 3. Removal of segments not in a shortest path. Of note, while steps 1–3 are shown sequentially (for better illustration), ADAG completes them in one step. 4. Removal of short end segments. 5. Merging adjacent segments. Note that adjacent segments in a GFA overlap (30 nt) so the length of the merged segment is shorter than the sum of the original segments. Bottom panel. Example of path retention in real GFA. **A** Original GFA from MetaCherchant with many short segments and loops. **B** An inset that zooms in GFA to show a complexity of the original assembly. **C** Final GFA after running ADAG to reduce the overall complexity and remove loops

Figure 5 illustrates the process of the ADAG algorithm on an example GFA wherein the ADAG simplified the overall complexity of the graph and removed loops. For this work, the path search is limited to the neighborhood of 2000 bp around the seed. This provides enough assembly to evaluate the genomic environment around the ARG yet ensures that all relevant paths can be annotated through homology search in a practical time frame. First, only segments that are part of the shortest path from any end segments are kept (Fig. 5, steps 1–3). Then, the GFA is further pruned by removing any end segments shorter than 250 bp, as these often represent very small spurious assembly branches (Fig. 5, step 4). Finally, all consecutive adjacent segments, except for the seed segments, are merged into new continuous segments Fig. 5, step 5). Figure 5A–C shows an example of this procedure on an actual GFA.

To perform all steps of GFA analysis and refinement described above, a new R package called gfaTools was developed within the environment of R version 4.0 + [23]. The package is distributed together with the MGS2AMR pipeline.

#### Genomic sequence homology search

Once the paths in each GFA are finalized, the MGS2AMR pipeline runs a sequence homology search of the path segments against the NCBI nucleotide database using the BLAST + software package [16]. All non-seed segments >  = 250 and seed fragments >  = 75 bp are used (e.g., Fig. 2B). Different GFA may have overlapping segments when their respective ARG are in close proximity to each other in the assembled sequence. This primarily occurs for plasmids or ARG cassettes [24]. To avoid performing homology search multiple times for GFA with overlapping segments, all segments are compared for sequence identity using the *cluster_fast* algorithm from the Usearch package [21]. Sequences are considered “identical” when they share 99% sequence identity and differ no more than 2.5% in length.

We used BLAST + settings optimized for long, high-scoring matches to any bacterial genome or plasmid (Table 1). After the sequence homology search, each GFA segment receives the number of matches to bacterial species accompanied by a set of alignment quality scores (e.g., bit score, coverage, and sequence identity) or zero if no matches are found.

**Table 1:** BLAST + settings used in MGS2AMR

Parameter	Value	Comment
megablast		Megablast is optimized for rapid homology search of nucleotide sequences with expected high sequence identity
taxidlist	All bacterial NCBI taxa ID	The nucleotide database is masked to include bacterial sequences only. The full list of IDs was obtained from the NCBI taxonomy database with Entrez filter “txid2[Organism:exp]” (*n* = 515,103 at the time of writing)
word_size	64	The performance of BLASTn in recovering a bacterial host was consistent across word sizes 32, 48, and 64. As the longer word size contributes to faster searches, 64 was selected
max_hsps	3	
qcov_hsp_perc	50	
perc_identity	75	
max_target_seqs	500	The initial BLAST + results are limited to the top 500 matches. However, when a particular segment matches 500 times with identical bit scores across bacteria, the sequence homology search is repeated with the identity and coverage set to the maximum value of the first run, with homology hits to return = 5000. This ensures that all top-scoring bacteria are represented in the results

#### Annotation by origin

In the final step of the MGS2AMR pipeline, the paths in the GFA and the segment homology search results from BLASTn are combined to annotate the detected ARG with any matching bacterial species. First, all homology hits annotated as *uncultured* or *mixed bacteria* are removed while matches to plasmids are assigned an extra label to distinguish them from genomic matches.

The homology search results are used to check if any of the detected ARG are duplicates (Additional file 1: Figure S2). If two or more ARG segments from different GFA overlap in the same bacterium (or plasmid), these GFA likely represent the same ARG and only the one with the highest bit score is kept. Similar logic is applied to verify if the distance between a segment and the seed ARG in the GFA is the same as the distance between their respective matches in a genome/plasmid. If not, the segment match is likely a false positive and is discarded.

**Fig. 6:**
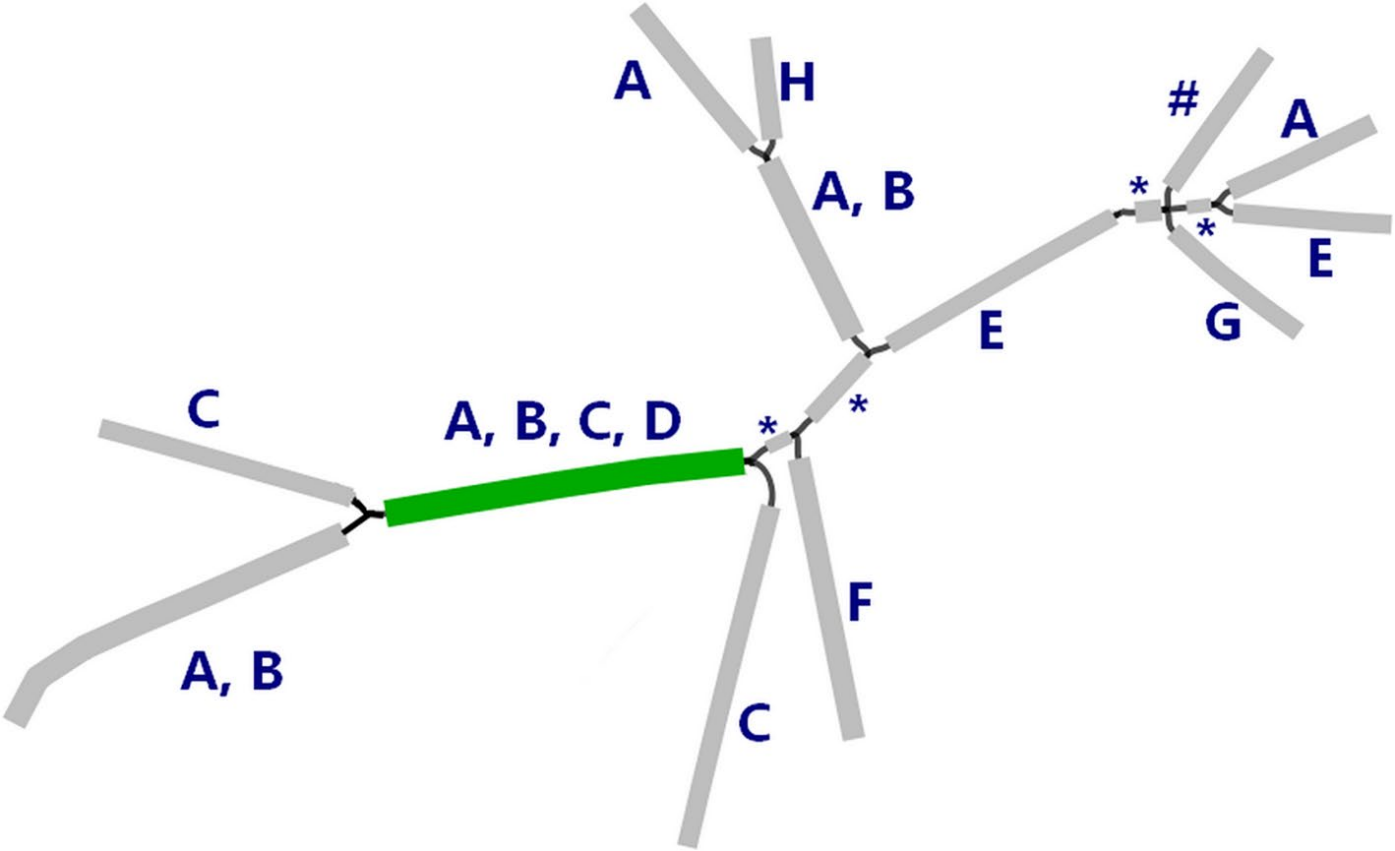
Schematic example of annotation of GFA segments after BLASTn. The green segment refers to the seed ARG and gray to the assembled environment. Letters **A**–**G** represent different bacterial strains matching the segments. Segments denoted by “*” are shorter than the segment cutoff and were not used in the homology search. “#” denotes that no significant homology hits were found. Hence, only strains **A**, **B**, **C**, and **D** are retained in the given example

Next, paths are annotated by the origin (Fig. 6). A bacterium is considered a match only if it is found in a path through consecutive matching segments starting from the seed ARG. Short segments not used for homology search (below cutoff, see previous section; denoted by ‘*’ in Fig. 6) are assumed to match the origin if the larger adjacent segments have a match. Paths are annotated at the strain level, i.e., segments have the same NCBI taxon ID.

Finally, the path score for each retained bacterial strain in the GFA is calculated. For the *N* segments in a path, let *C* be the segment coverage and *B* the corresponding bit-score computed by BLASTn. The total path score *Q* for the bacterium is as follows:

2 $$Q={\sum }_{i= 1}^{N}{B}_{i}\times {C}_{i}$$

The resulting annotation is a list of all candidate bacterial strains (i.e., accession numbers) that match in the GFA together with their total path score, the path score of the extension (i.e., excluding the seed ARG), the path length, and the path k-mer count. All data, including intermediate files, are saved in a user-defined output folder. Additionally, the MGS2AMR tool saves all results, metadata, and logs in an SQLite database, which allows not only easy data access but also resumption of the pipeline from intermediate stages in the case of unexpected issues. Additional details on the setup and configuration can be found in the MGS2AMR documentation.

### MGS2AMR explorer application

The output generated by the MGS2AMR pipeline contains a list of detected ARG associated with certain bacterial strains as the possible origin. However, all these strains are not necessarily present in a given microbiome, as one GFA path can potentially match multiple organisms in the vast NCBI nucleotide database. MGS2AMR keeps all matches, though the highest score should denote the best match for a particular ARG. The number of matches per ARG also depends on the quality of the assembly and subsequent processing, as long singular paths will generate more specific results compared to complex or fragmented assemblies. The results can be refined, e.g., by removing the likely false positives, through imposing specific cutoffs or downstream statistical analyses that can vary depending on the intended application. To gain more insights into the results, we designed an application to interactively visualize the MGS2AMR output.

MGS2AMR explorer is a R-Shiny application, distributed with the MGS2AMR package, that can be either run locally or installed on a server and accessed through an internet browser. The annotation data generated by MGS2AMR can be imported by either connecting to a MGS2AMR database or importing individual reports. The application allows the user to browse through the results and evaluate the detected ARG and matching bacteria.

### Benchmarking the MGS2AMR pipeline

Benchmarking is focused on recovering pathogen-specific AMR characteristics from simulated emerging intestinal infections caused by 6 clinically important bacteria: *Acinetobacter baumannii*,* Enterobacter sp*.,* Enterococcus faecium*,* Escherichia coli*,* Klebsiella pneumoniae,* and *Pseudomonas aeruginosa*. All listed bacteria are known to be major contributors to the global burden of AMR on human health [1]. The metadata on AMR (ARG and laboratory-derived AB resistance profile) were collected from the Pathogen Detection Project [25]. This online resource from NCBI reports ARG presence in isolates as reported by the AMRFinderPlus tool [5] together with laboratory results of clinical AMR testing. Isolates with ARG marked by AMRFinderPlus as “complete,” “partial,” “partial end of contig,” or “mistranslation” were considered in this study. All HTS files corresponding to the samples were downloaded from the NCBI Sequence Reads Archive using the SRA toolkit [26]. The SEQ2MGS tool [27] was used to combine sequencing reads from different existing FASTQ files in proportion to the desired final relative abundance producing a realistic simulation of the metagenome sequencing experiments. By mixing real microbiome samples with well-characterized isolates, the MGS2AMR pipeline can be assessed for the detection of a mixed-in pathogen while challenged with the full complexity of real MGS data.

For all bacteria of interest, sequencing experiments were randomly chosen from the Pathogen Detection Project. The healthy metagenomes were chosen from control samples used in published metagenomics studies across different continents. The latter ensures the benchmarking data represents the wide variety of healthy intestinal flora, which is influenced by many factors such as diet, geographical location, or genetic background. The original MGS files can vary greatly in size due to sequencing depth. For this work, the total genomic content was set to be between 1.5 and 4.5 Gbp. For all 6 species, 50 unique isolates were sampled and used 4 times. Isolates were randomly mixed into one of 100 backgrounds with a relative abundance between 1 and 10%. Although there is no definitive relative abundance at which intestinal infections can become clinically relevant, studies have shown that the range can be large, and sometimes only a few percent appears to be enough to cause illness [28]. Each background MGS sample was used 12 times. The complete list of the 1200 samples generated by SEQ2MGS can be found in the supplemental validation data details (Additional file 3).

MGS2AMR performance is evaluated on two criteria: the detection of the mixed-in bacterium and detection of the associated ARG (using the Pathogen Detection Project metadata as a gold standard). For this work, the results are evaluated at the bacterial genus level and differences in ARG alleles are ignored (e.g., CTX-M-16 and CTX-M-129 are considered the same gene). Extension from the ARG indicates an increased likelihood that the ARG is truly associated with the bacterium, which is quantified by the path score (a higher path score indicating higher confidence in the association). Since the isolate-associated ARG is known in a given simulated microbiome sample, recall and precision for the mixed-in bacterium can be calculated. Instances in which the true host shares a top score with other hosts are counted as true positives. However, the pipeline’s output reports all top-scoring ARG-host pairs. Additionally, we use the “ARG recall” metric to evaluate the sensitivity of the pipeline to detect the mixed-in ARG irrespective of the assignment of their origin. For example, if the mixed-in bacterium has 5 distinct ARG and all are detected by MGS2AMR, but only 4 are assigned to the original bacterium (the fifth ARG annotated differently), the bacterial recall is 80%, but the ARG recall is 100%.

### Application of MGS2AMR output to clinical AMR prediction

To demonstrate a potential application of the MGS2AMR pipeline output, we further processed the results generated from the 1200 samples (used to validate the tool) to predict the clinical AMR of the mixed-in bacterium (phenotypes known) to a set of specific AB.

Eight independent prediction models (one for each AB of interest) were built using a similar methodology described in detail in our previous work on phenotypic AMR prediction from sequenced bacterial isolates [7]. The AB considered for this work are ampicillin, cefepime, gentamicin, meropenem, tetracycline, tobramycin, trimethoprim-sulfamethoxazole, and vancomycin. The models were built species agnostic, i.e., no species information was used during the training and thus phenotypic AMR prediction can be made for all 6 bacteria of interest using the same model.

The data (bacterial geno- and phenotypes) to train, validate, and test the XGBoost models was sourced from the Pathogen Detection Project and supplemented with the PATRIC database to increase the number of available antibiograms [25, 29]. A total of 1739 isolates (across the 6 bacteria) with both geno- and phenotype information were collected. Table 2 summarizes the total number of samples available per antibiotic with the number of unique genes per relevant AMR class. As in our previous work, the XGBoost algorithm was chosen to train the models as it works well with sparse datasets, is less prone to overfitting, and provides the option to report important features of the model. As these models are merely used to demonstrate the possible application of the MGS2AMR pipeline and are not part of it, an in-depth analysis of the models or comparison of different machine learning methodologies for AMR prediction is outside of the scope of this work, but has been the topic of related research [6, 7].

**Table 2:** Instances used for building AMR XGBoost prediction models per antibiotic

Antibiotic	Class	Samples	ARG	S	R
Ampicillin	Beta-lactam	1376	28	57	1319
Cefepime	Beta-lactam	720	29	240	480
Gentamicin	Aminoglycoside	1550	52	560	990
Meropenem	Beta-lactam	1072	31	550	522
Tetracycline	Tetracycline	1333	7	446	887
Tobramycin	Aminoglycoside	1258	49	482	776
Trimethoprim-sulfamethoxazole	Sulfonamide	1356	3	329	1027
Vancomycin	Glycopeptide	95	7	15	80

Class represents the class of the antibiotic. Samples is the total number of samples available for training and testing the XGBoost models. ARG is the number of unique ARG found across these samples (and used as input). S and R are the numbers of samples susceptible or resistant to the AB, respectively, as defined by the clinical laboratory metadata (antibiogram)

Each model, one per AB, takes a binary vector of ARG detected (present = 1, absent = 0) as an input and predicts clinical AMR to the AB as a 2-class problem (susceptible = 0, resistant = 1). To ensure biological consistency between genotype input and phenotype output, only ARG belonging to the AB class of the resistance model was considered. For example, the model for gentamicin resistance prediction only considers aminoglycoside-related ARG and ignores ARG from other AMR classes that might be present.

For each model, 70% of the data was used for training, with the remaining part split into 40% validation data for tuning the hyperparameters of the models, and 60% testing data to evaluate the performance. The XGBoost objective was set to “Binary logistic” with a maximum tree depth of 3, a total of 500 iterations with an early stopping after 150 iterations when no improvement in learning occurred. All XGBoost models were built in R using the XGBoost library [23].

The metrics for performance evaluation on the test set include precision, recall, accuracy, and the Matthew correlation coefficient (*MCC*, Eq. 3).

3 $$MCC= \frac{\left(TP\times TN\right)-(FP\times FN)}{\sqrt{(TP+FP)(TP+FN)(TN+FP)(TN+FN)}}$$

Once the 8 models were built, the performance on their respective test sets (ARG vectors from pure isolate bacteria) was compared to the performance of known MGS2AMR genotypes (i.e., mixed in bacterium) recovered from the 1200 samples used to validate the pipeline. For this to work, MGS2AMR output first needed to be converted to ARG input vectors that could be supplied to the XGBoost models. ARG with no assembled extension was ignored if at least one other detected ARG had a path score *S* ≥ 3500. The path scores of all remaining ARG were normalized (Eq. 4).

4 $$\widehat{S}=\frac{log({S}_{i})}{max(log(S))}$$

The normalized values range between 0 and 1, with 0 referring to the absence of the ARG in the bacterium and 1 as the highest chance of ARG presence. The same metrics used to evaluate the XGBoost model performance on the test set are used to evaluate the performance of the processed output of the MGS2AMR pipeline.

## Results

### Summary of benchmark data

**Fig. 7:**
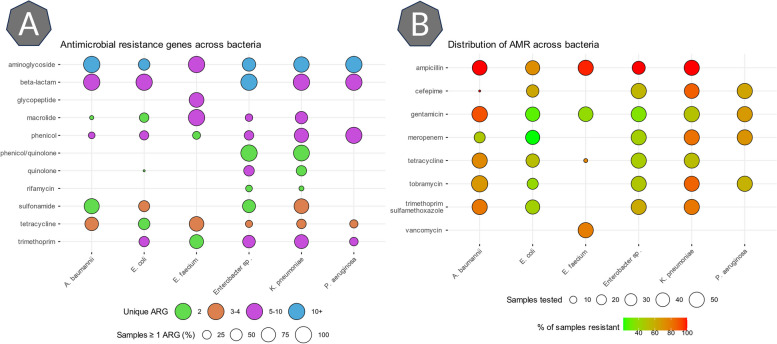
Distribution of ARG and AMR across the bacteria used to validate the pipeline. **A** Overview of the ARG present across bacteria used to validate the pipeline. The ARG are grouped by the AB class per bacterium. The size of the circle indicates the percentage of samples with at least one ARG belonging to the AB class, and the color indicates how many unique ARG (ignoring alleles) are present across all samples. **B** Overview of the resistance to 8 different antibiotics present across the bacteria used to validate the pipeline. The size of the circle indicates the number of samples in which antibiogram data on the antibiotic was available. The color indicates the percentage of all samples resistant to the antibiotic

Figure 7 summarizes the ARG reported in the metadata from the Pathogen Detection Project by AB class. A total of 152 unique genes were present across the 300 bacteria (50 for each of the 6 species) used for generating the partially annotated metagenomes. ARG belonging to the aminoglycoside and beta-lactam class have the largest overall presence (circle size in Fig. 7A). The top 3 ARG (ignoring alleles) present in over 100 bacteria each were blaOXA (beta-lactam resistance), fosA (fosfomycin resistance), and sul1 (sulfonamide resistance). Other common ARG families found are related to resistance to aminoglycoside antibiotics (e.g., aac(6’), ant(3’’), aph(3’), aph(6’)), trimetroprim antibiotics (e.g., dfrA), phenicol antibiotics (e.g., catA), tetracyclines (e.g., tet(A), tet(M)), sulfonamides (e.g., sul1, sul2), and many beta-lactams (e.g., blaADC, blaCTX-M, blaEC, blaPDC, blaSHV, blaTEM). A full list of all genotypes and phenotypes can be found in the supplemental validation data details (Additional file 3).

When the isolated bacteria were mixed into the healthy intestinal metagenomes by SEQ2MGS the average RA was 5.5% (± 1.7%). The generated metagenomic sequencing data were on average 2.86 Gbp (± 0.725) in size.

### GFA processing and homology search

**Fig. 8:**
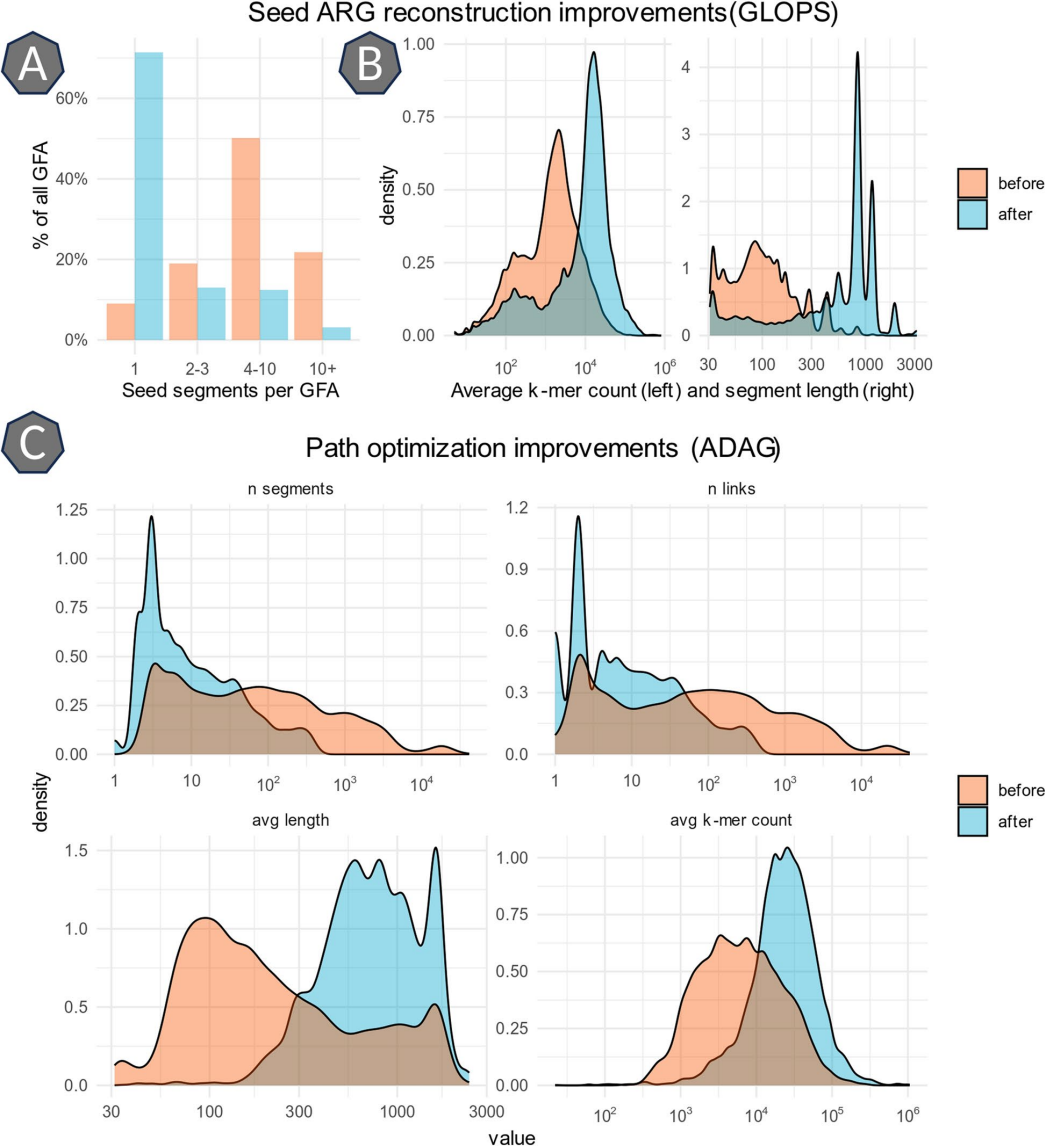
ARG assembly from metagenomic data improved by GLOPS and ADAG algorithms. **A** Reduction of the total number of seed segments after GLOPS. **B** Increase in seed segment length and k-mer count after GLOPS. **C** Path optimization using ADAG resulting in the reduction of the total number of segments/links in the GFA, with an increase in segment length and k-mer count. Note that all density plots have a log-scale *x*-axis. Sequence homology search using BLASTn on the segments constituting the final GFA paths returned at least one match with coverage of 97% (± 9%) and identity of 99.7% (± 1%) for 92% segments (818 ± 475 nt). This indicates that the GFA paths generated and filtered by the implemented steps represent real, biologically meaningful sequences

The first step in the pipeline, MetaCherchant assembly, yielded 697 (± 379) ARG assemblies on average per sample. In the next step, after applying the GLOPS algorithm, the number of ARG segments was reduced to a single segment in the majority of cases (Fig. 8A) while the length and *k*-mer count of the segments increased (Fig. 8B). After ARG reconstruction and filtering, the average number of ARG became 27 (± 10) per sample. Across all 1200 metagenomes, 1290 unique ARG were detected, of which 152 (12%) belonged to any of the six bacteria of interest. All other ARG were recovered from bacterial species present in the metagenomes used to generate the background sequencing data.

Once detected, ARG assemblies were further processed by the ADAG algorithm to find the top-scoring assembly paths emanating from the reconstructed ARG. As with ARG reconstruction, this again dramatically improved the length of segments (4.3 fold median increase) and *k*-mer counts (4.1 fold median increase) in the GFA as can be seen in Fig. 8C, resulting in an overall reduction of the total number of segments and links in each GFA (~ sevenfold decrease for both).

### ARG recovery and bacterial annotation

**Fig. 9:**
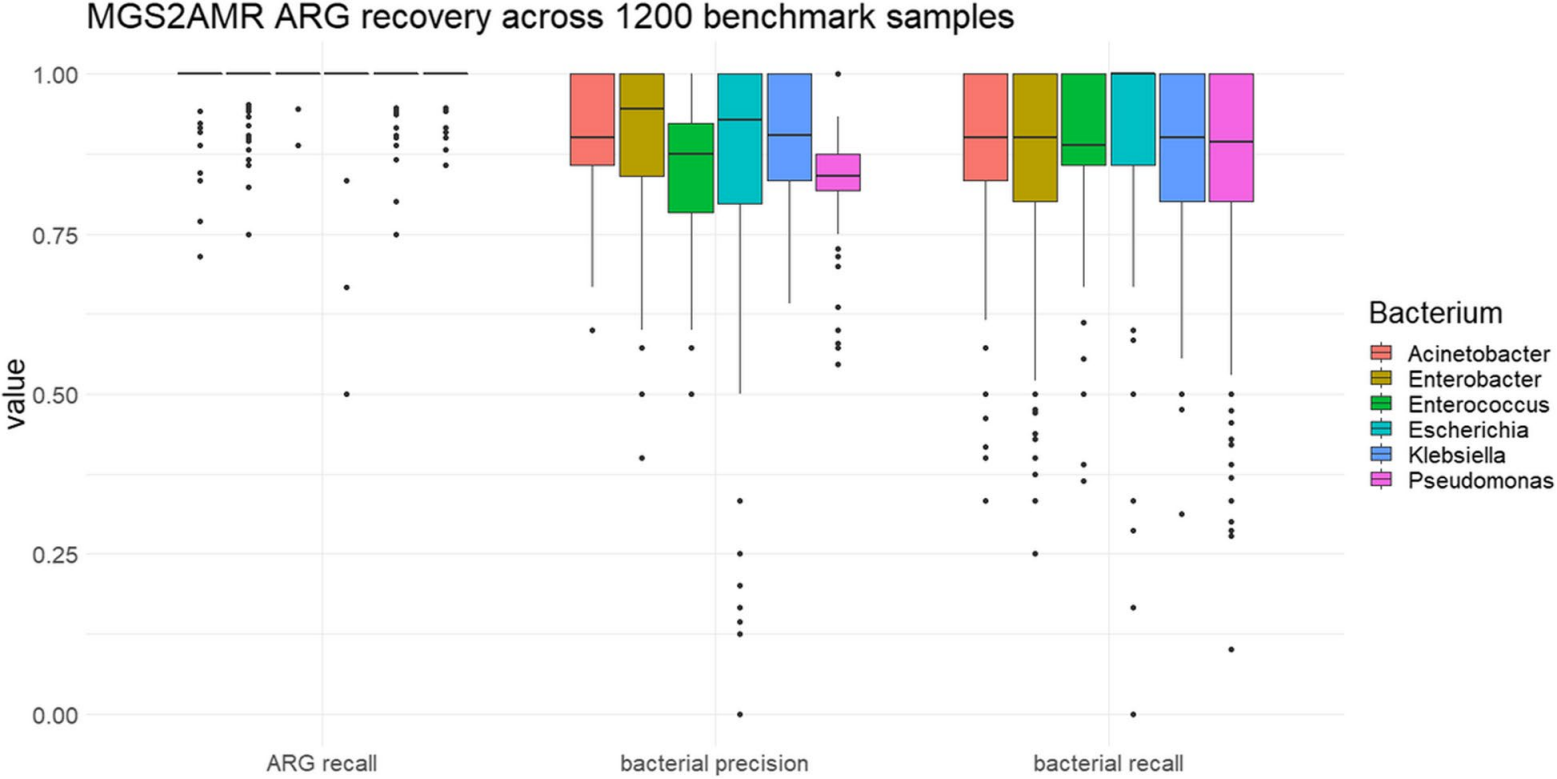
Performance of MGS2AMR on bacterial ARG recovery. Bacterial recall and precision are evaluated on ARG recovered from the mixed-in species from each of the 1200 metagenomes. The ARG recall represents the recovery of the ARG regardless of correct genus annotation or path score. Each boxplot represents the distribution of the given metric across the 200 samples per bacterium used for validation

ARG recall, defined as the percentage of the mixed-in *isolate* ARG detected in the microbiome sample, regardless of species, relative abundance, or background, is 98.9% (± 4.0%). When evaluating only the ARG correctly associated with the bacterium of interest, the recall is 87.5% (± 12.9%) with a precision of 87.5% (± 15.8%). Figure 9 shows the stratification of these metrics by bacterium.

Next, we assessed the metagenomics backgrounds in which the bacteria were mixed in for each sample. As outlined in the “Methods” section, all these backgrounds originated from healthy intestinal microbiomes with an unknown mix of bacteria and possible ARG. All output associated with the mixed-in bacterium was removed. The remaining results were evaluated at the bacterial genus level and summarized in Table 3 that lists the top 10 most commonly detected genera across all samples. All genera are known to be commensal to the human intestinal flora. As each of the 100 background metagenomes from healthy individuals was used 12 times, each time with different mixed-in isolates, the background species detected should be consistent across samples. This was quantified by the Bray–Curtis dissimilarity with an average dissimilarity of 0.36 (± 0.12) [30]. This shows MGS2AMR detects the same species consistently in samples known to be similar.

**Table 3:** Top bacterial genera found in the background metagenomes

Bacterial genus	Average total path score	Prevalence (%)
*Bacteroides*	21,193.82	88
*Barnesiella*	13,564.08	91
*Riemerella*	12,962.68	87
*Lachnospiraceae*	12,665.01	89
*Prevotella*	12,599.36	92
*Proteus*	12,248.33	76
*Enterobacter*	12,243.81	83
*Parabacteroides*	11,924.01	85
*Clostridium*	11,409.35	92
*Streptococcus*	11,208.43	85

The sample background is defined as bacterial genera present in the results of MGS2AMR after excluding the genus of the mixed-in bacterium. The total path score is the sum of all ARG path scores for a particular genus in a given sample. The average path score is used to rank overall genus presence across all analyzed samples. The prevalence is the percentage of samples reportedly containing the genus

When an ARG is detected by MGS2AMR, the respective assembly paths may match multiple bacteria. The higher the path score (Eq. 2), the more likely the match. The paths for the ARG known to belong to the mixed-in bacterium were top-scored in 98.6% (± 6.9%) cases. Table 4 shows the breakdown of the percentage when the correct match has the highest score by a bacterium.

**Table 4:** Correct ARG association with a bacterial genus based on the assembly path score

Genus	Top score (%)^a^	Plasmid (%)^b^	Odds ratio^c^
*Acinetobacter*	94.9	25.7	1.133
*Enterobacter*	83.6	59.4	1.452
*Enterococcus*	97.4	47.1	0.760
*Escherichia*	88.2	66.2	1.257
*Klebsiella*	89.3	60.8	1.402
*Pseudomonas*	88.2	9.1	3.288

^a^The percentage of cases in which an ARG known to belong to the mixed-in bacterium has the top path score assigned by MGS2AMR

^b^The percentage of cases where ARG are estimated to originate from plasmids

^c^The odds ratio an ARG originates from a plasmid or not when the known genus is not the top prediction

In 27.9% of cases, multiple bacteria share the highest path score for a particular ARG (i.e., ambiguity in bacterial assignment). However, in 70.8% of these cases, the match is labeled as a plasmid. The latter are known to have high sequence similarities between species complicating unique assignments [31]. In other words, when the ARG known to belong to the mixed-in bacterium does not yield the top score for that genus, they are more likely to be of the plasmid origin. This is shown by the odds ratio in Table 4 which divides the number of times an ARG that was not assigned the top-score for the known mixed-in bacterium when it was predicted to be on a plasmid versus when it was predicted to be of genomic origin.

### MGS2AMR Explorer

**Fig. 10:**
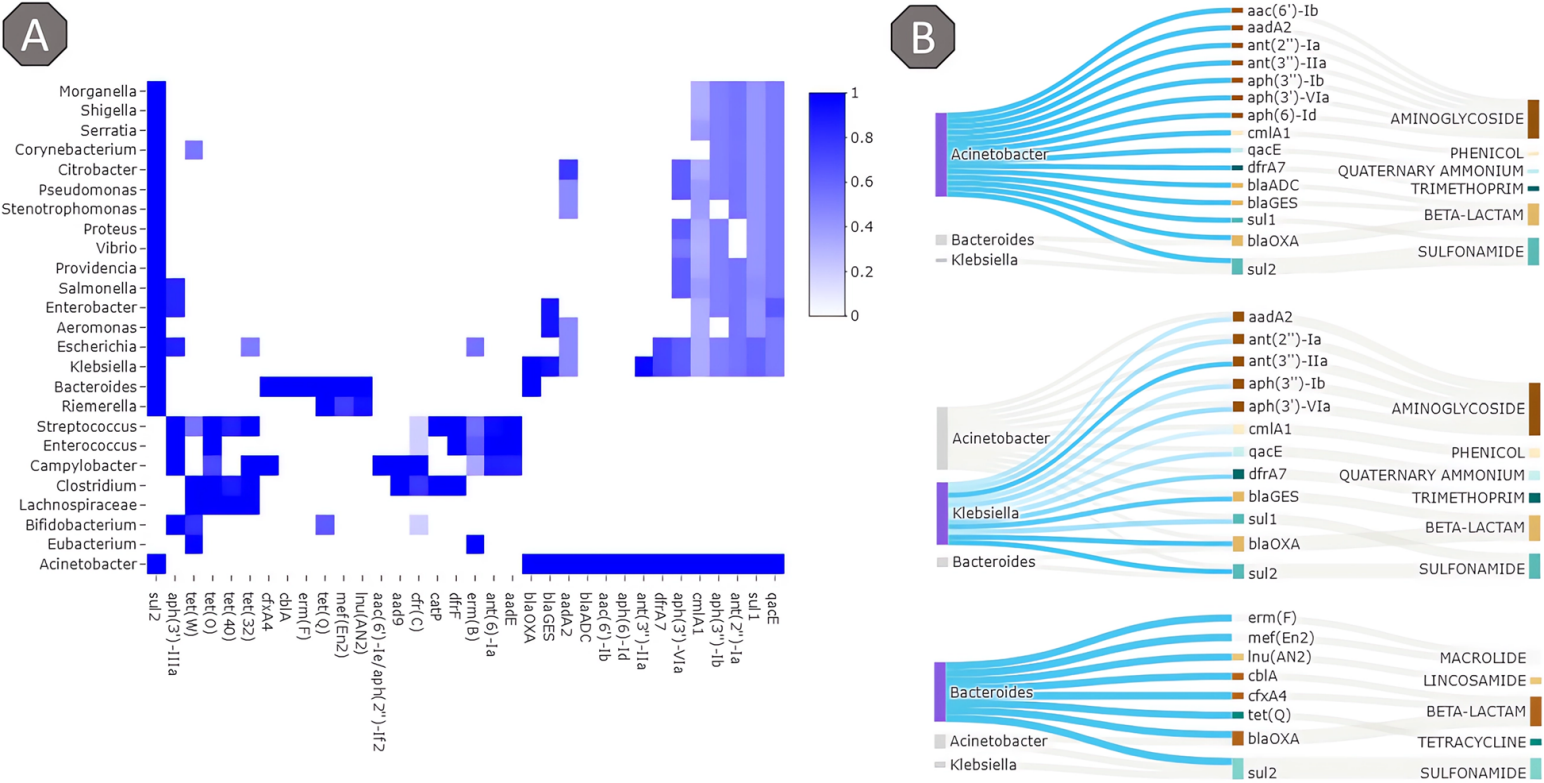
Example of the MGS2AMR Explorer visualization. **A** A heatmap of detected ARG and annotated bacteria, colored by the min–max normalized score (1 represents the top score, 0—no association determined). **B** Detailed exploration of specific bacteria and associated ARG. The blue-colored lines denote the genus in focus, with gray lines representing other top-scoring bacteria that match a particular ARG. Lighter blue shades are non-top-scoring matches. The image shows the alternative views of the same report upon changing the focus across the determined genera. Top: *Acinetobacter* is likely to be present as all ARG are top scoring with only two (blaOXA and sul2) having a potential association with another bacterium. Middle: *Klebsiella* is probably not present in the sample as all ARG are also present in *Acinetobacter,* and only 1 ARG (sul2) shares a top score (i.e., scores for all other ARG are lower than those for *Acinetobacter*). Bottom: *Bacteroides* has 6 top-scoring ARG that are not contested, so it likely is present in the sample

To get a better overview of the bacteria and ARG annotated by MGS2AMR, we developed the MGS2AMR explorer application. The results can be stratified by various parameters and combined in visualizations that provide insights. Figure 10A shows a heatmap of the top species detected in a sample known to contain *Acinetobacter baumannii*. Some ARG like blaADC are uniquely assigned to the bacterium. Others, like sul1 or ant(2’’)-Ia, have a top score assigned to the bacterium, but other bacteria also have matches though with lower scores. In some cases, like sul2 however, there is a large list of bacteria with (near) top scores, of which *Acinetobacter* is only one. This MGS2AMR Explorer visualization facilitates a conclusion that *Acinetobacter* is very likely to be truly present, as it has multiple ARG all with top scores. This is further visualized in the Sankey diagrams shown in Fig. 10B. In these plots, when focusing on a specific bacterium (purple/blue color), only bacteria with equal or higher scores for one or more ARG (gray color) are shown. The *Acinetobacter* plot shows very little overlap with other bacteria, whereas the focus on *Klebsiella* shows a large overlap with *Acinetobacter* suggesting *Klebsiella* to be likely a false positive. Both *Bacteroides* and *Acinetobacter* appear to be present, but blaOXA and sul2 genes cannot easily be disambiguated. By providing all the associated data and visualizations, the user can be better informed to decide how to further process the data and make specific calls.

While MGS2AMR Explorer provides valuable insights into the MGS2AMR output, the tool does not modify the data. This ensures that the output can be used for a variety of subsequent downstream applications.

### Example of using MGS2AMR output in predictive machine learning models

To showcase the potential downstream analyses using MGS2AMR output, we converted the genotypes of the recovered mixed-in bacteria (with known AMR phenotypes) from the 1200 samples into inputs for independently developed XGBoost machine learning models that predict clinical AMR based on a set of ARGs.

Table 5 shows the performance of the XGBoost models on their respective test sets and the comparison to the performance of the prediction when MGS2AMR genotypes were used. Although slightly lower, the prediction performance on MGS2AMR input is on par with the performance on the models’ test set data (derived from pure isolates). This suggests that MGS2AMR can effectively recover specific bacteria and their ARG from a large metagenome with enough accuracy to be used in a prediction model expecting input from a single bacterium and yield clinically relevant results.

**Table 5:** Performance of the 8 AMR prediction models on their respective test sets and MGS2AMR output

Antibiotic	Precision	Recall	Accuracy	MCC
Ampicillin	1.00/0.98	1.00/0.99	1.00/0.97	0.94/0.74
Cefepime	0.91/0.85	0.80/0.78	0.78/0.74	0.46/0.37
Gentamicin	0.93/0.73	0.95/0.89	0.92/0.81	0.83/0.63
Meropenem	0.86/0.74	0.74/0.78	0.78/0.74	0.57/0.48
Tetracycline	0.79/0.61	0.99/0.94	0.86/0.75	0.75/0.57
Tobramycin	0.95/0.76	0.99/0.97	0.96/0.84	0.91/0.70
Trimethoprim-sulfamethoxazole	0.97/0.86	0.95/0.93	0.93/0.86	0.81/0.70
Vancomycin	1.00/0.97	1.00/1.00	1.00/0.98	1.00/0.95

The first value is the performance on the XGBoost model test set (isolates only), and the second value is the performance on the MGS2AMR processed output (ARG recovered from the metagenomics data)

## Discussion

The MGS2AMR pipeline detects ARG and their possible origin within the metagenome sequencing data. This in silico bacterial recovery mimics laboratory isolation, culturing, and sequencing of bacteria from a microbiome sample, allowing for the evaluation of bacterial AMR directly from stool samples. The pipeline bypasses several hurdles of in vitro bacterium cultivation. First, bacteria have different growth requirements, such as the composition of the culture medium, aerobic vs anaerobic conditions. Some will not even grow at all under laboratory conditions. Second, cultivation is a time-consuming process that can take several days to weeks depending on the species. This is especially critical when patients with bacterial infections need a timely antibiotic treatment. Finally, the in vitro AMR analysis of metagenome samples would require isolating and testing all unique bacteria, while MGS2AMR works directly with the metagenomic sequencing data.

Both MetaCherchant and BLASTn were used in our MGS2AMR pipeline; however, the actual innovation stems from the use of two novel algorithms, namely GLOPS and ADAG, to optimize and annotate the genomic assembly paths within the raw GFA that resulted in generating accurate results. First, the GLOPS algorithm provides a way to reconstruct the ARG present in the data. An important advantage of GLOPS compared to simply counting the various pieces of seed ARG used in the assembly is that GLOPS takes into account the interspersed non-seed segments. Such a gene reconstruction step reduces the number of seed segments per GFA, with just a single, long segment of DNA (Fig. 8A, B). In this work, there were hundreds of cases where less than 75% of the seed segments were used in the assembly, but a whole gene was recovered after the GLOPS reconstruction. This reduces the pool of potential ARG candidates from hundreds (raw MetaCherchant output) to just a few dozen. However, the detection of ARG alone does not suffice to uniquely associate them with a bacterium. Many ARG can be found across different species, especially when residing on plasmids. The ADAG algorithm evaluates the assembly surrounding the ARG to aid in species annotation and to extract the most probable paths from the GFA, effectively creating much longer (4.1 fold on average), and fewer segments surrounding the ARG, also improving the sequence homology search results (Fig. 8C).

The results of 1200 tests (Fig. 9) show a very high ARG recall rate, indicating that MGS2AMR is very sensitive even at a low relative abundance of the bacterium. There are still some reasons why the detection of ARG may fail. One is related to uncertainty in the reference metadata from AMRFinderPlus (Pathogen Detection Project) used as a gold standard. For example, the ere(A) gene, when reported by AMRFinderPlus, was only detected in about half the samples processed by MGS2AMR. However, the former had labeled this ARG as “partial” or “partial end of contig” in 75% of the isolates. According to the AMRFinderPlus reference, this means that the coverage of the ARG was between 50 and 90%, denoting uncertainty in its presence. This example highlights that ARG detection is still an active area of research even in isolated bacteria. False positive calls can also be related to the presence of mosaic ARG in the Antimicrobial Resistance Reference Gene Database. Mosaic genes have not evolved from one ancestral gene, but rather contain the combined fragments of different genes. For example, the tetracycline resistance genes tet(O/M/O), tet(O/32/O), tet(W/32/O), and tet(O/W/O) are combinations of tet(M), tet(O), tet(W), and tet(32) [32]. When the latter, “simple” ARG were detected by the pipeline, the mosaic ARG would often be returned as well. None of the used isolates in the tested samples contained mosaic tetracycline ARG, but in 14% of the cases when a tetracycline resistance gene was detected, one or more mosaic ARG would be reported as well. While this lowers precision, in practice however, these ARG still represent the same class of AMR, which is less problematic than detection of ARG from the non-related AMR classes. High bacterial recall and precision (Fig. 9) are also encouraging, given the bacterium has a maximal relative abundance of 10% in any of the analyzed samples, and only 37% (± 17%) of the total number of ARG detected in any sample belong to the isolate.

MGS2AMR will assign ARG top scores to the correct genus in the vast majority of the cases (Table 4, first column). Even when the bacterium was not assigned the top score for an ARG known to be present, it still has a nearly top score (Table 4, second column). It is important to note, however, that not all bacteria listed in the MGS2AMR output are actually present in the microbiome, as the output lists all species with any match in the nucleotide database after homology search of the GFA paths. If the assembly and homology search were perfect, only the top scores should be considered (i.e., one bacterium per ARG), and the rest could be ignored as false positive matches. However, a strict cutoff like that is not recommended since closely related species and suboptimal assembly could assign the incorrect bacteria with top scores while the true positives are very close in score. Additional file 1: Figure S3 shows how certain bacteria detected in any of the 1200 samples are often associated with the actual ones mixed into the benchmarking samples.

Interestingly, when the performance of the models on the MGS2AMR dataset is broken down by bacterium instead of antibiotic, performance on predicting *P. aeruginosa* antibiotic resistance was lowest among organisms tested (Additional file 2: Table S1). Further analysis of *P. aeruginosa* resistance by antibiotic (Additional file 2: Table S2) shows that cefepime and meropenem models yield lower prediction power (MCC 0.14 and 0.09, respectively), whereas gentamicin and tobramycin perform much better (MCC 0.52 and 0.57, respectively). This stark difference can be explained by the fact that beta-lactam AB (cefepime and meropenem) in *Pseudomonas* requires porins to enter the bacterial cell whereas aminoglycosides do not. Porins are membrane channels, and not considered specific ARG, but mutations can affect their expression or permeability thus conferring indirect resistance [33]. Since porins are not present in the NCBI ARG database, MGS2AMR does not evaluate them. This explains why the performance of MGS2AMR is high for *P. aeruginosa* (i.e., when known ARG are present, they are detected), but the prediction of phenotypical AMR is lower. This alternative mechanism of resistance through porins highlights one of the limitations of MGS2AMR. Given the pipeline’s reliance on the curated ARG database, any genes not represented in the database will not be detected. This extends to the AB resistance mechanisms that rely on processes other than the simple presence of ARG, including the expression level of a porin channel in the example above or point mutations in various AB target genes associated with increased AMR. Future work could expand MGS2AMR by including point mutation mechanisms, though detection of a single polymorphism in a metagenome is even more challenging than gene detection and subsequent bacterial annotation. This will be particularly complex in instances where AMR-related mutations are present in otherwise highly conserved structures like the bacterial ribosome. Using the ribosomal genes as a seed for MetaCherchant would result in matches to numerous bacteria, and differentiation between species with or without a particular point mutation would be very challenging.

Another limitation of working with metagenomes focused on AMR analysis comes from the inherent unknowns present in the data. It is impossible to culture all bacteria present in a metagenome. Bioinformatics pipelines all rely on tailored experiments which are limited in scope or on simulated data for validation. This work is no exception; however, by using the SEQ2MGS tool, the amount of artificial data was kept to a minimum by using real sequencing data for both the background metagenome and the isolate mixed in. Though the pipeline could only be validated on the recovery of the isolate, the species present in the background would still be reported since MGS2AMR detects any species in sufficient relative abundance. Although full validation of the background species with respective ARG content is not possible, all metagenome backgrounds were selected from healthy participants serving as controls in various metagenomics studies. Table 3 shows that, when excluding the mixed in pathogens, the bacteria detected in the backgrounds belong to known commensals of the intestinal flora.

As the pipeline heavily relies on the NCBI nucleotide database for bacterial annotation, one must take into account its inherent biases. This database has a higher presence of specific species, especially when relevant to human health. In effect, these bacteria will have a higher representation in the database increasing the chance of any match when homology searches are conducted. This phenomenon could also explain some of the cases with lower precision seen in Fig. 9. Most of the bacteria evaluated in this work are very well studied (i.e., human pathogens) and thus should have high representation in the nucleotide database. If a less studied commensal species (i.e., background) contains ARG that can also be found in the pathogens studied, they might be falsely associated with that pathogen instead. This underscores that, for now, MGS2AMR is most useful for detecting AMR of bacterial species that are both well studied and in sufficient abundance in the metagenome (e.g., emerging infection). MGS2AMR is not a metagenomic tool for taxonomic classification (tools that identify all species present in a metagenome), but rather aimed at evaluating the most abundant species containing one or more ARG of interest. However, the tool effectively identifies important species without the need for separate taxonomic classification.

In theory, the concepts that underlie the presented computational MGS2AMR pipeline could be applied to any set of genes that might be of interest in metagenomic analysis. By changing the list of seed genes submitted to MetaCherchant, these genes would become the center of assembly and thus subsequent cluster forming and species annotation. Additional research outside of this paper’s scope is needed to validate the expansion to genes unrelated to AMR. One factor that makes this pipeline suitable for AMR evaluation is the specific presence or absence of genes (i.e., ARG). Only species containing the gene (or highly similar genomic regions) will assemble around it, with subsequent evaluation validating its presence and potential host. In contrast, highly conserved genes used as input would likely end up mapping back to many species with low specificity. This still is the case for many ARG shared across species, and MGS2AMR has several built-in steps that are taken to reduce the false positive rate.

The adoption of MetaCherchant as the metagenome assembler within the MGS2AMR pipeline is pivotal in achieving optimal speed and efficiency. In the supplementary materials (Additional file 4), we provide a comprehensive breakdown of the MGS2AMR pipeline into its individual steps, with a thorough assessment of the required computational resources, including runtime and peak memory usage. Within the four pipeline steps, the most significant consumption of time and RAM is attributed to BLAST + . Additional file 1: Figure S4 illustrates the speed of the MGS2AMR pipeline, indicating a runtime of approximately 1150 s (around 20 min). The innovative approach of MetaCherchant, which involves assembly centered around seeds of interest (ARGs), greatly contributes to the swift execution of the initial assembly phase. It is noteworthy that the runtime corresponds with the file size, as expected. Interestingly, the time dedicated to homology search appears to show no correlation with the original file size.

While alternative pipelines could potentially be constructed using different metagenome assemblers, such as metaSPAdes [34], these alternatives frequently engage in assembling the entire metagenome. This, unfortunately, results in a substantial amount of time being spent on irrelevant regions unrelated to our specific objective of detecting and annotating ARGs. Additionally, it is important to recognize that ARGs or similar seed sequences do not hold a central role in such assembly process, which necessitates the use of supplementary tools for their identification before subsequent analysis. In the same supplementary document (Additional file 4), we present a comparative analysis between MGS2AMR and an alternative workflow. In this alternative approach, we replace the first MetaCherchant step and subsequent assembly analysis (including GLOPS and ADAG) with metaSPAdes [34] and DIAMOND [35]. While the alternative approach is feasible and could potentially benefit from further refinement steps not explored here, its notable drawback lies in its slower pace. The pipeline requires a considerably longer runtime, with metaSPAdes contributing to the majority of the processing time, and subsequently results in lower ARG recall rates (Additional file 2: Table S4). On average, the completion time for the pipeline extends to approximately 90 min, with the largest file taking around 2 h. It is important to observe that in the case of the alternative pipeline, runtime scales proportionally with the file size due to the extended assembly process.

## Conclusions

In summary, MGS2AMR provides a novel way of exploring antimicrobial resistance in a microbiome specimen. In addition to detecting ARG, it also associates these genes with bacteria of potential origin. The pipeline returns the assemblies of identified ARG with extension into their surrounding genomic regions. This allows for the bacterium-specific evaluation of antimicrobial resistance based on the metagenomic sequencing data offering many potential applications, ranging from early prediction of the AMR profile of bacterial infections to evaluating the presence and origin of ARG across specific bacteria in a microbiome sample.

### Supplementary Information

**Additional file 1: Fig. S1.** Resolving shortest paths with loops in GFA. Green segment is the start and end of the loop. 1. Loop that begins and ends on the different sides of the start-segment. Resolved by generating two paths (A,B,C,D) and (A,D,C,B). Note that the sequence direction of A differs in two paths. 2. Loop that begins and ends on the same end of the start-segment. Resolved similar to Loop 1, but the direction of A is identical in both paths. 3. Hairpin loop with repeated segments A, B and C. Resolved by creating two paths (A,B,C,D,E,F) and (A,B,C,F,E,D). 4. Hairpin loop with different start- (A) and end- (H) segments. Resolved by removing all path data (G and H) after the repeated segment (C), reducing the problem to the hairpin loop in example 3 with the same solutions: (A,B,C,D,E,F) and (A,B,C,F,E,D). **Fig. S2.** Example of the evaluation of homology matches. The seed segments of ARG1 and ARG2 both match a reference genome at the same position, indicating they refer to the same ARG. The position of segment 4 in the reference genome does not align with the expected distance from the ARG as represented in the GFA of ARG 1 suggesting it likely represents a false positive match, and therefore will be excluded from further analysis. **Fig. S3.** Bacteria associated with the 6 bacteria used in validation. This heatmap shows which bacterial sequences (both genome or plasmid) also tend to score high when the known presence is one of the 6 used in validation. It reflects the uncertainty that comes with bacterial calling in metagenomics. **Fig. S4.** MGS2AMR run time and memory usage for 5 benchmarking samples. All tools were allowed to use up to 8 CPUs. The numbers 1 through 5 refer to the file ID in **Table S3.** The four main pipeline steps are denoted as follows: A. MetaCherchant (existing tool). B. The MetaCherchant output pre-processing for BLAST (novel R scripts). C. BLAST+ (existing tool) D. ARG annotation (novel R scripts). Note that the large leap in memory for BLASTn is nearly entirely explained by having to load the nucleotide database into memory (~150 GB).

**Additional file 2: Table S1.** Breakdown of the MGS2AMR output by genus. Details on XGBoost model performance on the dataset used to validate the MGS2AMR pipeline. **Table S2.** Breakdown of MGS2AMR output for *Pseudomonas*. Details on XGBoost model performance for *Pseudomonas.*

**Additional file 3:** Metadata utilized for the validation of the MGS2AMR pipeline. **ARG:** The list of all antimicrobial resistance genes (ARG) evaluated by the pipeline. **Backgrounds:** Information on metagenomic sequencing data from healthy individuals, sourced from previously published studies (control samples). These data serve as the background for the 1200 samples generated for validation. **GeneLinks:** The linkage between genotypic information and the ARG tables. **Genotypes:** Details on the antimicrobial resistance genotypes of all bacteria used in the generation of the 1200 validation samples. **Phenotypes:** Information on the antimicrobial resistance phenotypes exhibited by all bacteria used in the generation of the 1200 validation samples. SampleInfo: Details on the sequencing data used in the generation of the 1200 validation samples. **SEQ2MGS_1200:** Input parameters for SEQ2MGS to generate the 1200 validation samples.

**Additional file 4: Table S3.** Dataset used for benchmarking. Samples used to benchmark the MGS2AMR pipeline. **Table S4.** Comparison of the ARG annotation by MGS2AMR and the alternative pipeline. The performance of the MGS2AMR pipeline in comparison with the alternative (metaSPAdes/DIAMOND) pipeline.

## Data Availability

• All MGS2AMR pipeline scripts are hosted on https://github.com/pieterjanvc/mgs2amr with packaged releases available at https://github.com/pieterjanvc/mgs2amr/releases under the MIT license. Releases contain all required data (except external dependencies) to run the full pipeline. • Instructions for installing pipeline dependencies can be found in the tool’s documentation (readme.txt). The gfaTools R package, developed for this work, is distributed with the release and should be installed prior to running the pipeline. • The MGS2AMR Explorer Shiny application is also distributed with the releas and can be run independently on any MGS2AMR output. • The datasets analyzed during the current study are available in the Sequence Read Archive repository, https://www.ncbi.nlm.nih.gov/sra. All accession numbers can be found in the supplemental file Additional file 3 under the SEQ2MGS_1200 tab.

## Abbreviations

AB: Antibiotic
AMR: Antimicrobial resistance
ARG: Antibiotic resistance gene
GFA: Graphical fragment assembly
HTS: High-throughput sequencing
MGS: Metagenomic sequencing
NCBI: National Center for Biotechnology Information

## References

[1] Murray CJ, Ikuta KS, Sharara F, Swetschinski L, Aguilar GR, Gray A, et al. Global burden of bacterial antimicrobial resistance in 2019: a systematic analysis. The Lancet [Internet]. 2022 [cited 2022 Feb 3];0. Available from: https://www.thelancet.com/journals/lancet/article/PIIS0140-6736(21)02724-0/fulltext. 10.1016/S0140-6736(21)02724-0 PMC8841637 35065702

[2] Antibiotic Resistance Threats in the United States, 2019. Atlanta: GA: U.S. Department of Health and Human Services, CDC; 2019.

[3] Patel R, Fang FC (2018). Diagnostic stewardship: opportunity for a laboratory–infectious diseases partnership. Clin Infect Dis.

[4] Blair JMA, Webber MA, Baylay AJ, Ogbolu DO, Piddock LJV (2015). Molecular mechanisms of antibiotic resistance. Nat Rev Microbiol.

[5] Feldgarden M, Brover V, Gonzalez-Escalona N, Frye JG, Haendiges J, Haft DH (2021). AMRFinderPlus and the Reference Gene Catalog facilitate examination of the genomic links among antimicrobial resistance, stress response, and virulence. Sci Rep.

[6] Van Camp P-J, Haslam DB, Porollo A (2020). Bioinformatics approaches to the understanding of molecular mechanisms in antimicrobial resistance. Int J Mol Sci.

[7] Van Camp P-J, Haslam DB, Porollo A (2020). Prediction of antimicrobial resistance in gram-negative bacteria from whole-genome sequencing data. Front Microbiol.

[8] Ransom EM, Potter RF, Dantas G, Burnham C-AD (2020). Genomic prediction of antimicrobial resistance: ready or not, here it comes!. Clin Chem.

[9] Rózsa L, Apari P, Sulyok M, Tappe D, Bodó I, Hardi R (2017). The evolutionary logic of sepsis. Infect Genet Evol.

[10] Zhang C, Xiu L, Li Y, Sun L, Li Y, Zeng Y (2021). Multiplex PCR and nanopore sequencing of genes associated with antimicrobial resistance in Neisseria gonorrhoeae directly from clinical samples. Clin Chem.

[11] Wang M-Y, Geng J-L, Chen Y-J, Song Y, Sun M, Liu H-Z (2017). Direct detection of mecA, blaSHV, blaCTX-M, blaTEM and blaOXA genes from positive blood culture bottles by multiplex-touchdown PCR assay. Lett Appl Microbiol.

[12] Schmidt K, Stanley KK, Hale R, Smith L, Wain J, O’Grady J (2019). Evaluation of multiplex tandem PCR (MT-PCR) assays for the detection of bacterial resistance genes among Enterobacteriaceae in clinical urines. J Antimicrob Chemother.

[13] Virolle C, Goldlust K, Djermoun S, Bigot S, Lesterlin C (2020). Plasmid transfer by conjugation in gram-negative bacteria: from the cellular to the community level. Genes (Basel).

[14] Partridge SR, Kwong SM, Firth N, Jensen SO (2018). Mobile genetic elements associated with antimicrobial resistance. Clin Microbiol Rev.

[15] Olekhnovich EI, Vasilyev AT, Ulyantsev VI, Kostryukova ES, Tyakht AV (2018). MetaCherchant: analyzing genomic context of antibiotic resistance genes in gut microbiota. Bioinformatics.

[16] Camacho C, Coulouris G, Avagyan V, Ma N, Papadopoulos J, Bealer K (2009). BLAST+: architecture and applications. BMC Bioinformatics.

[17] The GFA Format Specification Working Group. Graphical Fragment Assembly (GFA) Format Specification [Internet]. GFA-spec. 2022 [cited 2022 Mar 1]. Available from: http://gfa-spec.github.io/GFA-spec/GFA1.html.

[18] Wick RR, Schultz MB, Zobel J, Holt KE (2015). Bandage: interactive visualization of de novo genome assemblies. Bioinformatics.

[19] Evans BA, Amyes SGB (2014). OXA β-Lactamases. Clin Microbiol Rev.

[20] Schöning U (1988). Graph isomorphism is in the low hierarchy. J Comput Syst Sci.

[21] Edgar RC (2010). Search and clustering orders of magnitude faster than BLAST. Bioinformatics.

[22] Dijkstra EW (1959). A note on two problems in connexion with graphs. Numer Math.

[23] R Core Team (2021). R: a language and environment for statistical computing [Internet]. R Foundation for Statistical Computing, Vienna, Austria; Available from: https://www.R-project.org/.

[24] Partridge SR, Tsafnat G, Coiera E, Iredell JR (2009). Gene cassettes and cassette arrays in mobile resistance integrons. FEMS Microbiol Rev.

[25] The NCBI Pathogen Detection Project. Bethesda (MD): National Library of Medicine (US), National Center for Biotechnology Information -https://www.ncbi.nlm.nih.gov/pathogens/ (Accessed 2022–05–01) [Internet]. [cited 2022 May 1]. Available from: https://www.ncbi.nlm.nih.gov/pathogens/.

[26] SRA Toolkit Development Team. SRA Toolkit [Internet]. NCBI - National Center for Biotechnology Information/NLM/NIH - http://ncbi.github.io/sra-tools/ (Accessed 2022–05–01); 2019 [cited 2022 May 1]. Available from: https://github.com/ncbi/sra-tools.

[27] Van Camp P-J, Porollo A (2022). SEQ2MGS: an effective tool for generating realistic artificial metagenomes from the existing sequencing data. NAR Genom Bioinform.

[28] Shimasaki T, Seekatz A, Bassis C, Rhee Y, Yelin RD, Fogg L (2019). Increased relative abundance of Klebsiella pneumoniae Carbapenemase-producing Klebsiella pneumoniae within the gut microbiota is associated with risk of bloodstream infection in long-term acute care hospital patients. Clin Infect Dis.

[29] Wattam AR, Abraham D, Dalay O, Disz TL, Driscoll T, Gabbard JL (2014). PATRIC, the bacterial bioinformatics database and analysis resource. Nucleic Acids Res.

[30] Legendre P, Legendre LFJ. Numerical Ecology. Elsevier Science; 1998.

[31] Redondo-Salvo S, Fernández-López R, Ruiz R, Vielva L, de Toro M, Rocha EPC (2020). Pathways for horizontal gene transfer in bacteria revealed by a global map of their plasmids. Nat Commun.

[32] Warburton PJ, Amodeo N, Roberts AP (2016). Mosaic tetracycline resistance genes encoding ribosomal protection proteins. J Antimicrob Chemother.

[33] Pang Z, Raudonis R, Glick BR, Lin T-J, Cheng Z (2019). Antibiotic resistance in Pseudomonas aeruginosa: mechanisms and alternative therapeutic strategies. Biotechnol Adv.

[34] Nurk S, Meleshko D, Korobeynikov A, Pevzner PA (2017). metaSPAdes: a new versatile metagenomic assembler. Genome Res.

[35] Buchfink B, Xie C, Huson DH (2015). Fast and sensitive protein alignment using DIAMOND. Nat Methods.

