# Supplementary figures and images for: MGS2AMR: a gene-centric mining of metagenomic sequencing data for pathogens and their antimicrobial resistance profile
Source: Microbiome. 2023 Oct 13;11:223. doi: 10.1186/s40168-023-01674-z (PMC10571262; doi:10.1186/s40168-023-01674-z)

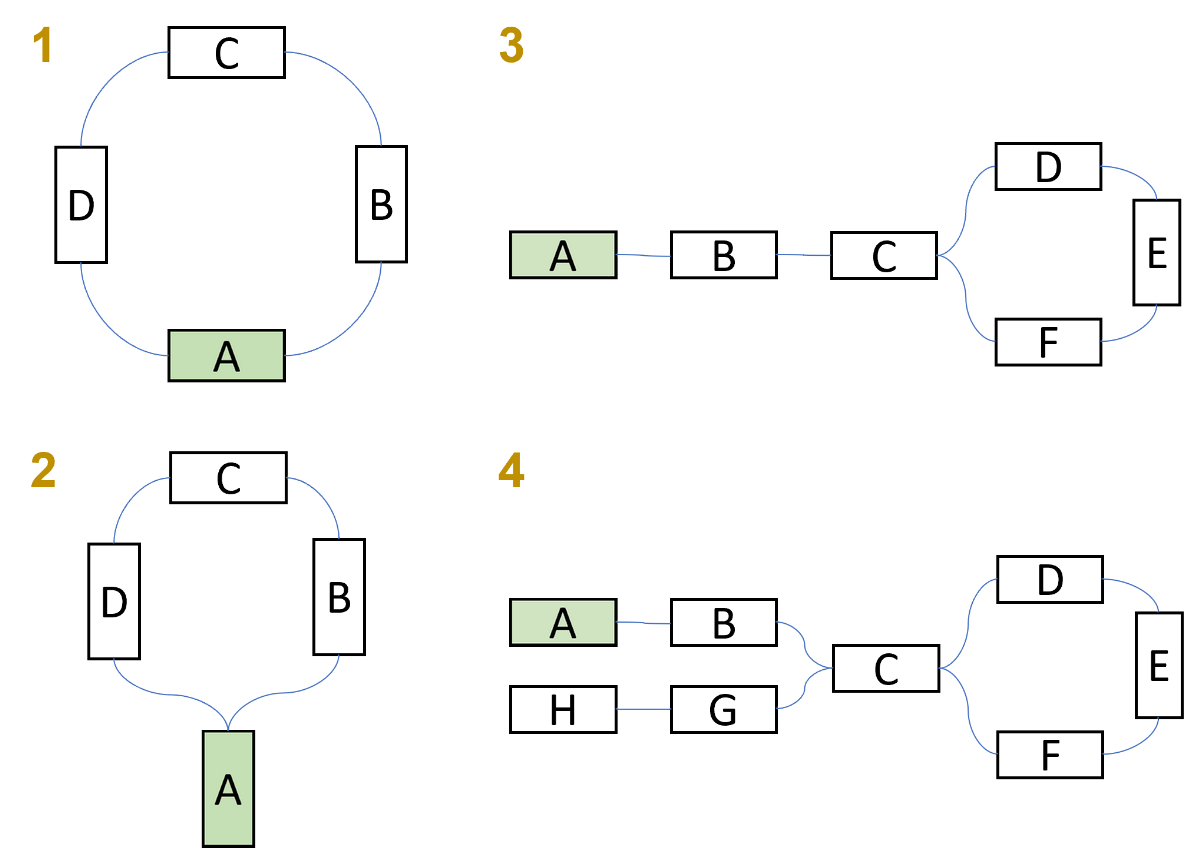

Supplement: Supplementary file 2 — Additional file 1: Fig. S1. Resolving shortest paths with loops in GFA. Green segment is the start and end of the loop. 1. Loop that begins and ends on the different sides of the start-segment. Resolved by generating two paths (A,B,C,D) and (A,D,C,B). Note that the sequence direction of A differs in two paths. 2. Loop that begins and ends on the same end of the start-segment. Resolved similar to Loop 1, but the direction of A is identical in both paths. 3. Hairpin loop with repeated segments A, B and C. Resolved by creating two paths (A,B,C,D,E,F) and (A,B,C,F,E,D). 4. Hairpin loop with different start- (A) and end- (H) segments. Resolved by removing all path data (G and H) after the repeated segment (C), reducing the problem to the hairpin loop in example 3 with the same solutions: (A,B,C,D,E,F) and (A,B,C,F,E,D). Fig. S2. Example of the evaluation of homology matches. The seed segments of ARG1 and ARG2 both match a reference genome at the same position, indicating they refer to the same ARG. The position of segment 4 in the reference genome does not align with the expected distance from the ARG as represented in the GFA of ARG 1 suggesting it likely represents a false positive match, and therefore will be excluded from further analysis. Fig. S3. Bacteria associated with the 6 bacteria used in validation. This heatmap shows which bacterial sequences (both genome or plasmid) also tend to score high when the known presence is one of the 6 used in validation. It reflects the uncertainty that comes with bacterial calling in metagenomics. Fig. S4. MGS2AMR run time and memory usage for 5 benchmarking samples. All tools were allowed to use up to 8 CPUs. The numbers 1 through 5 refer to the file ID in Table S3. The four main pipeline steps are denoted as follows: A. MetaCherchant (existing tool). B. The MetaCherchant output pre-processing for BLAST (novel R scripts). C. BLAST+ (existing tool) D. ARG annotation (novel R scripts). Note that the large leap in memory for BLAST [file 40168_2023_1674_MOESM1_ESM.zip › SupplementalFigureS1.png]

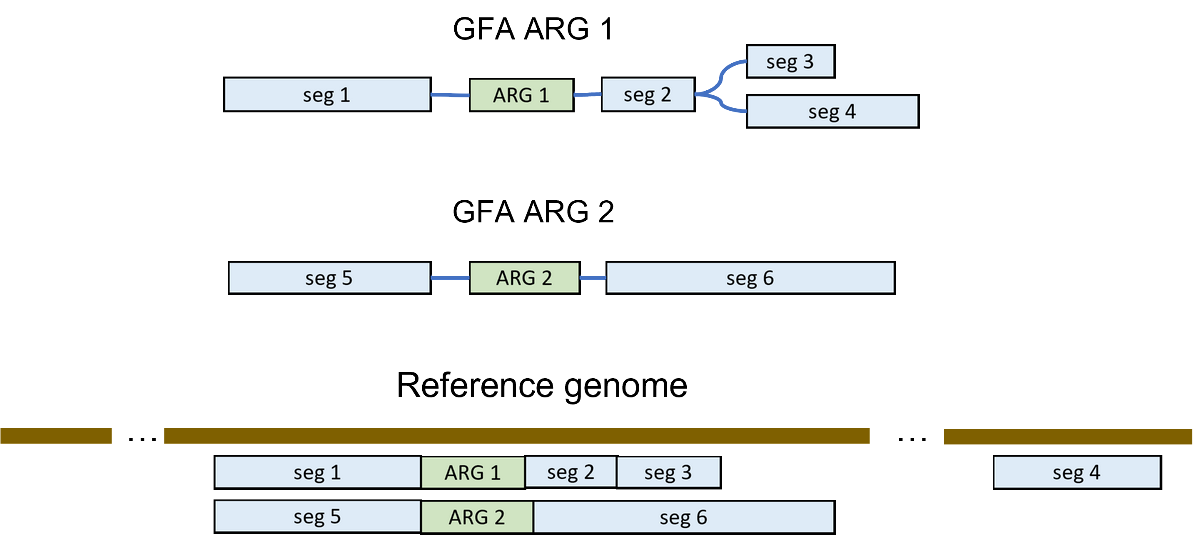

Supplement: Supplementary file 2 — Additional file 1: Fig. S1. Resolving shortest paths with loops in GFA. Green segment is the start and end of the loop. 1. Loop that begins and ends on the different sides of the start-segment. Resolved by generating two paths (A,B,C,D) and (A,D,C,B). Note that the sequence direction of A differs in two paths. 2. Loop that begins and ends on the same end of the start-segment. Resolved similar to Loop 1, but the direction of A is identical in both paths. 3. Hairpin loop with repeated segments A, B and C. Resolved by creating two paths (A,B,C,D,E,F) and (A,B,C,F,E,D). 4. Hairpin loop with different start- (A) and end- (H) segments. Resolved by removing all path data (G and H) after the repeated segment (C), reducing the problem to the hairpin loop in example 3 with the same solutions: (A,B,C,D,E,F) and (A,B,C,F,E,D). Fig. S2. Example of the evaluation of homology matches. The seed segments of ARG1 and ARG2 both match a reference genome at the same position, indicating they refer to the same ARG. The position of segment 4 in the reference genome does not align with the expected distance from the ARG as represented in the GFA of ARG 1 suggesting it likely represents a false positive match, and therefore will be excluded from further analysis. Fig. S3. Bacteria associated with the 6 bacteria used in validation. This heatmap shows which bacterial sequences (both genome or plasmid) also tend to score high when the known presence is one of the 6 used in validation. It reflects the uncertainty that comes with bacterial calling in metagenomics. Fig. S4. MGS2AMR run time and memory usage for 5 benchmarking samples. All tools were allowed to use up to 8 CPUs. The numbers 1 through 5 refer to the file ID in Table S3. The four main pipeline steps are denoted as follows: A. MetaCherchant (existing tool). B. The MetaCherchant output pre-processing for BLAST (novel R scripts). C. BLAST+ (existing tool) D. ARG annotation (novel R scripts). Note that the large leap in memory for BLAST [file 40168_2023_1674_MOESM1_ESM.zip › SupplementalFigureS2.png]

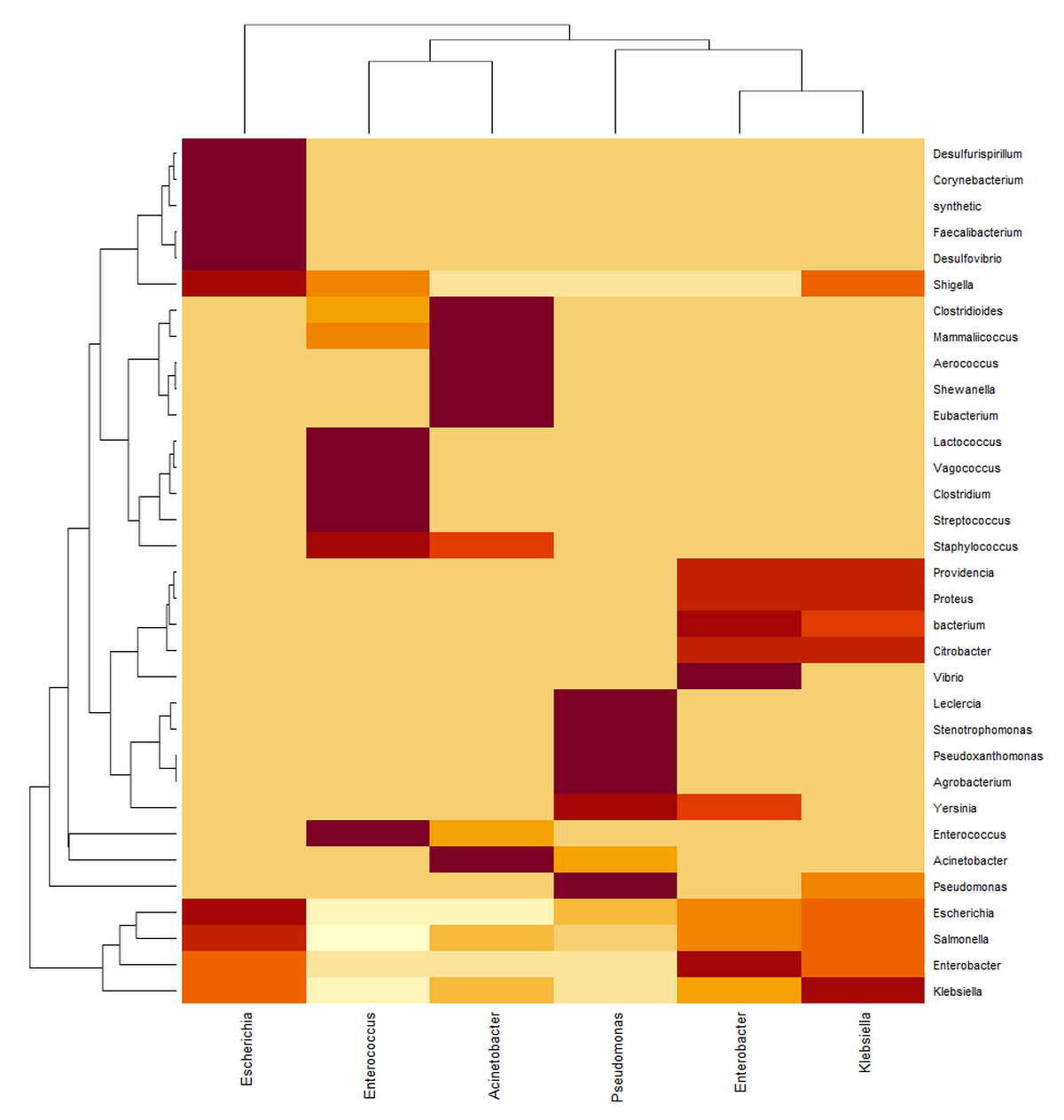

Supplement: Supplementary file 2 — Additional file 1: Fig. S1. Resolving shortest paths with loops in GFA. Green segment is the start and end of the loop. 1. Loop that begins and ends on the different sides of the start-segment. Resolved by generating two paths (A,B,C,D) and (A,D,C,B). Note that the sequence direction of A differs in two paths. 2. Loop that begins and ends on the same end of the start-segment. Resolved similar to Loop 1, but the direction of A is identical in both paths. 3. Hairpin loop with repeated segments A, B and C. Resolved by creating two paths (A,B,C,D,E,F) and (A,B,C,F,E,D). 4. Hairpin loop with different start- (A) and end- (H) segments. Resolved by removing all path data (G and H) after the repeated segment (C), reducing the problem to the hairpin loop in example 3 with the same solutions: (A,B,C,D,E,F) and (A,B,C,F,E,D). Fig. S2. Example of the evaluation of homology matches. The seed segments of ARG1 and ARG2 both match a reference genome at the same position, indicating they refer to the same ARG. The position of segment 4 in the reference genome does not align with the expected distance from the ARG as represented in the GFA of ARG 1 suggesting it likely represents a false positive match, and therefore will be excluded from further analysis. Fig. S3. Bacteria associated with the 6 bacteria used in validation. This heatmap shows which bacterial sequences (both genome or plasmid) also tend to score high when the known presence is one of the 6 used in validation. It reflects the uncertainty that comes with bacterial calling in metagenomics. Fig. S4. MGS2AMR run time and memory usage for 5 benchmarking samples. All tools were allowed to use up to 8 CPUs. The numbers 1 through 5 refer to the file ID in Table S3. The four main pipeline steps are denoted as follows: A. MetaCherchant (existing tool). B. The MetaCherchant output pre-processing for BLAST (novel R scripts). C. BLAST+ (existing tool) D. ARG annotation (novel R scripts). Note that the large leap in memory for BLAST [file 40168_2023_1674_MOESM1_ESM.zip › SupplementalFigureS3.png]

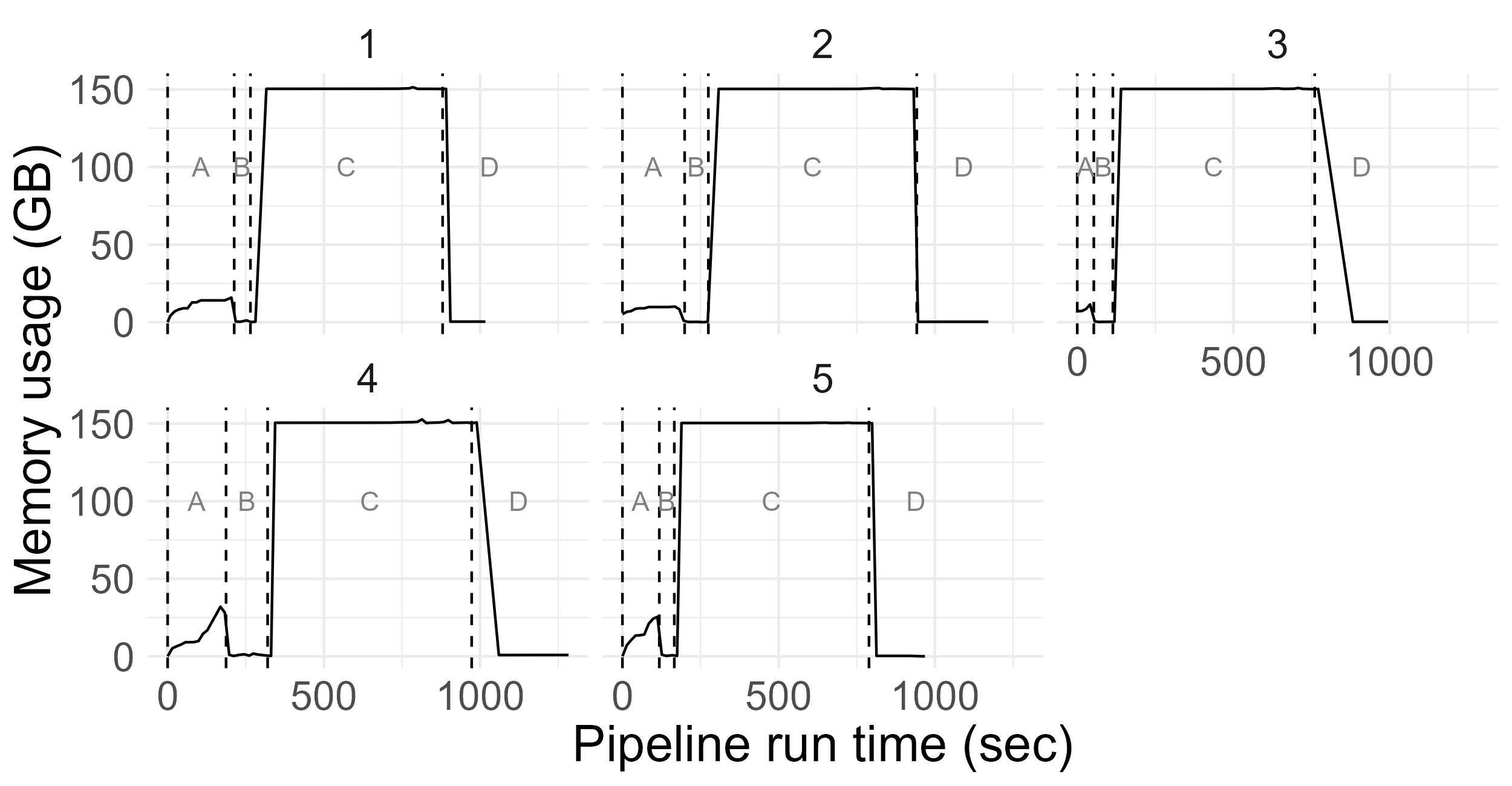

Supplement: Supplementary file 2 — Additional file 1: Fig. S1. Resolving shortest paths with loops in GFA. Green segment is the start and end of the loop. 1. Loop that begins and ends on the different sides of the start-segment. Resolved by generating two paths (A,B,C,D) and (A,D,C,B). Note that the sequence direction of A differs in two paths. 2. Loop that begins and ends on the same end of the start-segment. Resolved similar to Loop 1, but the direction of A is identical in both paths. 3. Hairpin loop with repeated segments A, B and C. Resolved by creating two paths (A,B,C,D,E,F) and (A,B,C,F,E,D). 4. Hairpin loop with different start- (A) and end- (H) segments. Resolved by removing all path data (G and H) after the repeated segment (C), reducing the problem to the hairpin loop in example 3 with the same solutions: (A,B,C,D,E,F) and (A,B,C,F,E,D). Fig. S2. Example of the evaluation of homology matches. The seed segments of ARG1 and ARG2 both match a reference genome at the same position, indicating they refer to the same ARG. The position of segment 4 in the reference genome does not align with the expected distance from the ARG as represented in the GFA of ARG 1 suggesting it likely represents a false positive match, and therefore will be excluded from further analysis. Fig. S3. Bacteria associated with the 6 bacteria used in validation. This heatmap shows which bacterial sequences (both genome or plasmid) also tend to score high when the known presence is one of the 6 used in validation. It reflects the uncertainty that comes with bacterial calling in metagenomics. Fig. S4. MGS2AMR run time and memory usage for 5 benchmarking samples. All tools were allowed to use up to 8 CPUs. The numbers 1 through 5 refer to the file ID in Table S3. The four main pipeline steps are denoted as follows: A. MetaCherchant (existing tool). B. The MetaCherchant output pre-processing for BLAST (novel R scripts). C. BLAST+ (existing tool) D. ARG annotation (novel R scripts). Note that the large leap in memory for BLAST [file 40168_2023_1674_MOESM1_ESM.zip › SupplementalFigureS4.png]
