# Supplementary material for: MGS2AMR: a gene-centric mining of metagenomic sequencing data for pathogens and their antimicrobial resistance profile
Source: Microbiome. 2023 Oct 13;11:223. doi: 10.1186/s40168-023-01674-z (PMC10571262; doi:10.1186/s40168-023-01674-z)
Supplement: Supplementary file 3 — Additional file 2: Table S1. Breakdown of the MGS2AMR output by genus. Details on XGBoost model performance on the dataset used to validate the MGS2AMR pipeline. Table S2. Breakdown of MGS2AMR output for Pseudomonas. Details on XGBoost model performance for Pseudomonas. [file 40168_2023_1674_MOESM2_ESM.docx]

Supplemental table S1 – Breakdown of the MGS2AMR output by genus

| Genus |  | recall | precision | accuracy | MCC |
| --- | --- | --- | --- | --- | --- |
| Acinetobacter |  | 0.91 | 0.94 | 0.88 | 0.65 |
| Enterobacter |  | 0.73 | 0.91 | 0.81 | 0.64 |
| Enterococcus |  | 0.93 | 0.9 | 0.88 | 0.69 |
| Escherichia |  | 0.74 | 0.89 | 0.84 | 0.68 |
| Klebsiella |  | 0.82 | 0.95 | 0.83 | 0.61 |
| Pseudomonas |  | 0.75 | 0.77 | 0.69 | 0.33 |

Supplemental table S2 – Breakdown of MGS2AMR output for *Pseudomonas*

| antibiotic | recall | precision | accuracy | MCC |
| --- | --- | --- | --- | --- |
| cefepime | 0.96 | 0.66 | 0.65 | 0.14 |
| gentamicin | 0.58 | 0.97 | 0.70 | 0.52 |
| meropenem | 0.87 | 0.72 | 0.67 | 0.09 |
| tobramycin | 0.58 | 0.96 | 0.74 | 0.57 |
