# Supplementary material for: MGS2AMR: a gene-centric mining of metagenomic sequencing data for pathogens and their antimicrobial resistance profile
Source: Microbiome. 2023 Oct 13;11:223. doi: 10.1186/s40168-023-01674-z (PMC10571262; doi:10.1186/s40168-023-01674-z)
Supplement: Supplementary file 5 — Additional file 4: Table S3. Dataset used for benchmarking. Samples used to benchmark the MGS2AMR pipeline. Table S4. Comparison of the ARG annotation by MGS2AMR and the alternative pipeline. The performance of the MGS2AMR pipeline in comparison with the alternative (metaSPAdes/DIAMOND) pipeline. [file 40168_2023_1674_MOESM4_ESM.docx]

Benchmarking MGS2AMR and comparison to an alternative pipeline

# Benchmarking dataset

Five sequencing files were semi-randomly selected from the pool of 1200 samples generated by SEQ2MGS and utilized in the evaluation of the MGS2AMR pipeline. These files were specifically chosen to encompass variations in file size, relative abundance, and resistance profiles of the spike-in pathogens of interest (Table S3).

**Supplemental table** **S3.** Dataset used for benchmarking.

| **ID** | **Spiked-in isolate**  **(Bacterium / SRA ID)** | **Background**  **(SRA ID)** | **File size**  **(fastq.gz)** | **Relative abundance** |
| --- | --- | --- | --- | --- |
| 1 | *E. faecium* (ERR1557083) | ERR2017415 | 3.48 GB | 4.1% |
| 2 | *P. aeruginosa* (SRR13302163) | ERR2017444 | 3.00 GB | 2.8% |
| 3 | *A. baumannii* (SRR3170776) | ERR3277283 | 0.93 GB | 5% |
| 4 | *K. pneumoniae* (SRR4065647) | ERR3277347 | 1.66 GB | 9.8% |
| 5 | *E. coli* (SRR13338366) | ERR2197823 | 1.26 GB | 3% |

# Resource utilization

As described in Methods of the main text (Figure 1), the MG2AMR pipeline has 4 major steps:

1. Detect the seed ARG and assemble genomic regions around them by MetaChechant
2. Analyze the MetaCherchant output by reconstructing ARG and building paths within the assemblies
3. Align the paths generated in step 2 to the nucleotide database using BLASTn
4. Combine the alignment output with the ARG and path information to annotate ARG with bacteria of potential origin

Steps 1 and 3 involve existing tools, namely MetaCharchant and BLAST+. On the other hand, steps 2 and 4 are innovative components specially crafted and executed in R for the purpose of this study. Detailed explanations of these novel elements can be found in the main text. For all five benchmarking samples listed in Table S3, we documented the time taken to complete each step and the memory usage associated with it. A visual representation of this data is available in Supplemental Figure S4. The benchmarking procedure for each file was consistently conducted on the same hardware configuration, utilizing 8 CPUs. This choice of hardware configuration enabled the tools to efficiently distribute computations in a parallel manner if feasible.


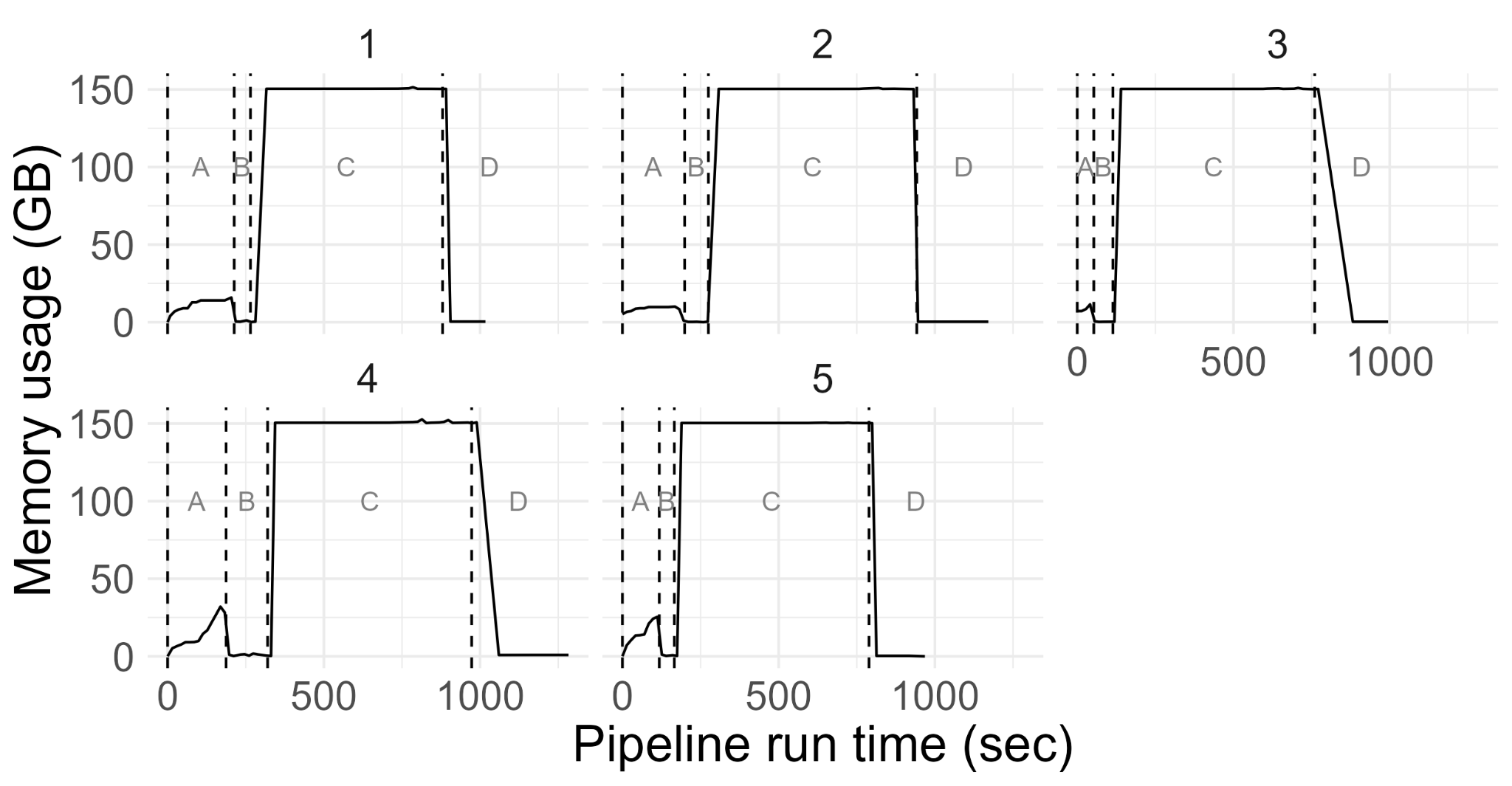


**Supplemental figure S4**. MGS2AMR run time and memory usage for 5 benchmarking samples. All tools were allowed to use up to 8 CPUs. The numbers 1 through 5 refer to the file ID in Table S3. The four main pipeline steps are denoted as follows: **A.** MetaCherchant (existing tool). **B.** The MetaCherchant output pre-processing for BLAST (novel R scripts). **C.** BLAST+ (existing tool) **D.** ARG annotation (novel R scripts). Note that the large leap in memory for BLASTn is nearly entirely explained by having to load the nucleotide database into memory (~150 GB).

# Performance comparison

For comparison, we developed an alternative pipeline in the following setup:

1. Metagenome assembly is performed by metaSPAdes
2. Any regions containing ARG are detected using DIAMOND
3. Custom R scripts analyze the metaSPAdes and DIAMOND output to decide on specific ARG presence and select relevant scaffolds with regions up to 5000 bp around any ARG (similar to step 2 in the MGS2AMR pipeline)
4. BLASTn is used to align the scaffolds to the nucleotide database to discover relevant bacteria (similar to step 3 in the MGS2AMR pipeline with identical settings)
5. Custom R scripts analyze the output and annotate ARG with specific bacteria (similar to step4 in the MGS2AMR)

The performance of the alternative pipeline, as measured by ARG recall (see "Benchmarking the MGS2AMR pipeline" in the main text), is compared to that of MGS2AMR using the same set of benchmarking data (Table S3). To ensure a fair comparison, a true positive is defined as a top-scoring ARG-bacterium combination that is present in the known spiked-in bacterium. A false positive, on the other hand, is any top-scoring ARG-bacterium combination that is not actually present in the known spiked-in bacterium. A false negative occurs when an ARG known to be present in the spiked-in bacterium is not detected or does not receive a top score (i.e., it is assigned to another bacterium). Table S4 provides the performance results of the both pipelines.

**Supplemental table S4.** Comparison of the ARG annotation by MGS2AMR and the alternative pipeline.

|  | **MGS2AMR** | | **Alternative pipeline** | |
| --- | --- | --- | --- | --- |
| **ID** | **ARG recall** | **Run Time** | **ARG recall** | **Runtime** |
| 1 | 80% | 1032s (0.28h) | 63% | 7338s (2.0h) |
| 2 | 77% | 1206s (0.34h) | 55% | 5600s (1.6h) |
| 3 | 75% | 1142s (0.32h) | 70% | 2955s (0.8h) |
| 4 | 83% | 1362s (0.38h) | 84% | 4942s (1.4h) |
| 5 | 100% | 965s (0.27h) | 72% | 6055s (1.7h) |
